# Dax1 modulates ERα-dependent hypothalamic estrogen sensing in female mice

**DOI:** 10.1038/s41467-023-38618-y

**Published:** 2023-05-29

**Authors:** Jose M. Ramos-Pittol, Isabel Fernandes-Freitas, Alexandra Milona, Stephen M. Manchishi, Kara Rainbow, Brian Y. H. Lam, John A. Tadross, Anthony Beucher, William H. Colledge, Inês Cebola, Kevin G. Murphy, Irene Miguel-Aliaga, Giles S. H. Yeo, Waljit S. Dhillo, Bryn M. Owen

**Affiliations:** 1 Institute of Biochemistry and Center for Molecular Biosciences Innsbruck, University of Innsbruck, Innsbruck, 6020 Austria; 2 Section of Investigative Medicine, Imperial College London, London, United Kingdom; 3 Institute of Clinical Sciences, Imperial College London, London, United Kingdom; 4 Department of Physiology, Development, and Neuroscience, Cambridge University, Cambridge, United Kingdom; 5 Medical Research Council Metabolic Diseases Unit, Wellcome-MRC Institute of Metabolic Science-Metabolic Research Laboratories, Cambridge University, Cambridge, United Kingdom; 6 Department of Histopathology and East Midlands & East of England Genomic Laboratory Hub, Cambridge University Hospitals NHS Foundation Trust, Cambridge, UK; 7 Section of Genetics and Genomics, Imperial College London, London, United Kingdom; 8 MRC London Institute of Medical Sciences, London, United Kingdom

**Keywords:** Neuroscience, Molecular biology, Reproductive biology, Endocrinology

## Abstract

Coupling the release of pituitary hormones to the developmental stage of the oocyte is essential for female fertility. It requires estrogen to restrain kisspeptin (KISS1)-neuron pulsatility in the arcuate hypothalamic nucleus, while also exerting a surge-like effect on KISS1-neuron activity in the AVPV hypothalamic nucleus. However, a mechanistic basis for this region-specific effect has remained elusive. Our genomic analysis in female mice demonstrate that some processes, such as restraint of KISS1-neuron activity in the arcuate nucleus, may be explained by region-specific estrogen receptor alpha (ERα) DNA binding at gene regulatory regions. Furthermore, we find that the *Kiss1*-locus is uniquely regulated in these hypothalamic nuclei, and that the nuclear receptor co-repressor NR0B1 (DAX1) restrains its transcription specifically in the arcuate nucleus. These studies provide mechanistic insight into how ERα may control the KISS1-neuron, and *Kiss1* gene expression, to couple gonadotropin release to the developmental stage of the oocyte.

## Abstract (web summary)

Estrogen controls female fertility in part via restraining or promoting kisspeptin (Kiss1)-neuron activity in the arcuate hypothalamic nucleus and the AVPV hypothalamic nucleus, respectively. Here the authors report that estrogen receptor alpha (ERα) interacts with the genome and the nuclear receptor co-repressor NR0B1 (DAX1) to manifest region-specific actions on *Kiss1* expression.

## Introduction

Ovarian estrogen production signals the developmental stage of the oocyte to hypothalamic nuclei. These nuclei, in turn, control the pulsatile output of gonadotropins from the pituitary. However, the mechanistic basis for how estrogen-sensing is conveyed to gonadotrophin-releasing hormone neurons is incompletely understood.

The estrogen receptor alpha (ERα, ESR1) is expressed in at least two regions of the hypothalamus that regulate female fertility; the arcuate nucleus, and the so-called ‘AVPV’ nucleus (the rostral periventricular area of the third ventricle, which contains both the periventricular nucleus and the anteroventral periventricular nucleus)^1–6^. Kisspeptin (KISS1)-expressing neurons of the arcuate hypothalamic nucleus are now firmly established as the gonadotropin-releasing hormone (GnRH) pulse-generator and are restrained by the negative feedback actions of estrogen^7–9^. In contrast, KISS1- neurons of the AVPV hypothalamus are stimulated by estrogen and are responsible for generating the mid-cycle LH-Surge^4,10–13^. Interestingly, *Kiss1* gene transcription appears to reflect the activity of the neuronal population, as it is repressed by estrogen in the arcuate nucleus and induced by estrogen in the AVPV nucleus^5,14–18^. These transcriptional effects are dependent on ERα^1–6^. However, while several mechanisms have been proposed^19^, it is currently not clear how ERα can orchestrate these opposing transcriptional effects on *Kiss1* in arcuate and AVPV nuclei. Furthermore, it is even less clear how a single nuclear receptor might mediate negative feedback by restraining the pulse frequency of arcuate KISS1-neurons.

We conducted a comparative survey of ERα DNA-binding in the arcuate and AVPV of female mice by chromatin immunoprecipitation followed by DNA sequencing (ChIP-seq). We found marked differences in ERα DNA-binding events in the arcuate and AVPV which pointed to differential functional outcomes driven by ERα activation. Specifically, our analysis revealed an enrichment of ERα-binding sites in genes that can modulate neuronal activity and pulse frequency under estrogen stimulation in the arcuate nucleus. In addition, we found that the *Kiss1* gene itself is uniquely regulated by ERα in these two nuclei and that the nuclear receptor co-repressor Dax1 is responsible, at least in part, for its restraint in the arcuate. Together, our studies provide mechanistic insight into how ERα may control the KISS1-neuron, and *Kiss1* gene expression, in order to couple gonadotropin release to the developmental stage of the oocyte.

## Results

### ERα binds to different genomic loci in the arcuate and AVPV nuclei that can coordinate KISS1 neuron function

The arcuate and AVPV nuclei of the hypothalamus are respectively responsible for translating the effects of estrogen-negative and positive feedback into changes in gonadotropin secretion^8,10^. However, how estrogen differentially affects the activity of these neurons, and in particular how it mediates negative feedback in the arcuate nucleus, is poorly understood. In order to gain insight into these processes, we first mapped genomic ERα binding sites in these locations by chromatin immunoprecipitation followed by DNA sequencing (ChIP-Seq). We used an estrogen-treatment paradigm in mice that allowed us to evaluate ERα activity at a time when negative feedback in the arcuate is occurring^20^.

**Fig. 1:**
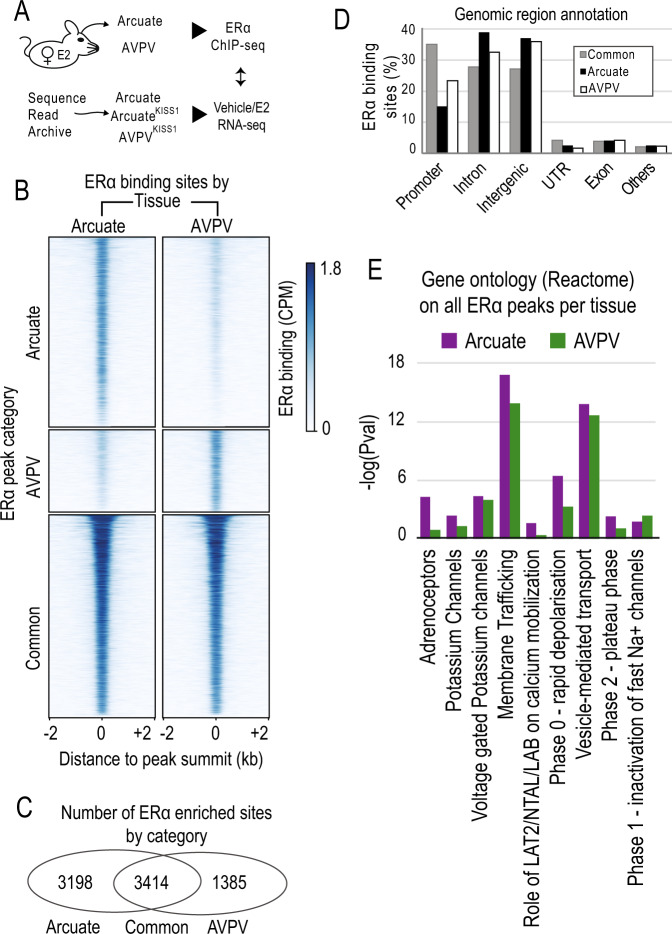
ERα binds to different genomic loci in the arcuate and AVPV nuclei. **A** Schematic representation of integrated analysis of ERα ChIP-seq data and nucleus-specific transcriptomic data. **B** ERα occupancy in identified by ChIP-seq peaks, categorized according to Arcuate- or AVPV-enrichment. **C** Venn diagram of ERα-enriched peaks in both locations. **D** Genomic annotation of ERα peaks separated by enrichment category. **E** Selected GO-term enrichment *p*-values in genes proximal to ERα peaks.

Our results show that ERα binding sites diverge substantially between the two regions analysed (Fig. 1A–C, Supplementary Figure 1A). Indeed, we were surprised to observe that over 50% of the binding sites were differentially enriched between the arcuate and the AVPV (Fig. 1C). These differential binding sites were mostly located to introns or intergenic regions, but were also found in promoters, suggesting nucleus-specific gene regulation by ERα involves both proximal and distal (enhancer) elements^21^ (Fig. 1D, Supplementary Data 1, Supplementary Data 2). Together, these findings demonstrate broad differences in ERα-binding in the arcuate and AVPV under estrogen stimulation.

In order to elucidate possible functional consequences driven by arcuate- or AVPV-specific ERα binding sites, we performed gene ontology (GO) term analysis (Supplementary Data 3) on our ChIP-seq data^22^. We found that terms relating to *Membrane Trafficking*, *Vesicle-mediated Transport*, *Rapid Depolarisation*, and *Adrenoreceptors* were enriched in both arcuate and AVPV nuclei (Fig. 1E). Intriguingly, the enrichment of genes bound by ERα and associated with these neurotransmitter processing and release GO terms was generally higher in the arcuate compared to the AVPV nucleus (Fig. 1E). This raises the possibility that under estrogen stimulation, ERα may have a greater impact in *Kiss1* neuronal activity through gene expression in the arcuate than it does in the in the AVPV. In order to explore this possibility, we re-analysed transcriptomic data from arcuate^17^ and AVPV KISS1 neurons (AVPV^KISS1^)^18^ in response to estrogen stimulation and explored ERα-peak proximal-genes (Supplementary Data 4, Supplementary Data 5 and Supplementary Data 6). We found that the expression of many genes associated with enriched GO terms were specifically altered by estrogen in the arcuate nucleus (Supplemental Fig. 2). Taken together, these analyses demonstrate that ERα binding in the arcuate regulates genes in response to estrogen that could modulate KISS1-neuronal function via neurotransmitter processing and release.

### ERα binds in the proximity of genes that coordinate KISS1-neuron pulsatility in the arcuate nucleus

**Fig. 2:**
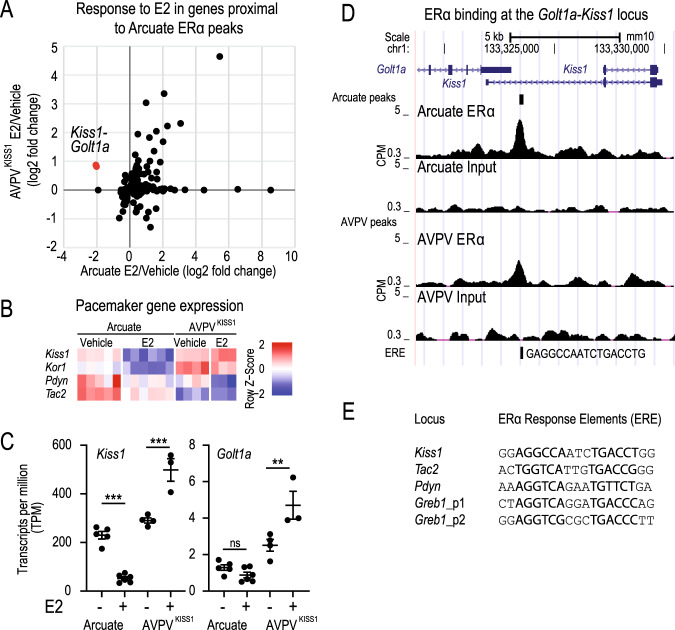
ERα binds in the proximity of genes that coordinate *Kiss1*-neuron pulsatility in the arcuate nucleus. **A** Genes found in proximity to ERα binding sites and affected by estrogen in the arcuate nucleus. **B** Expression of *Kiss1*, *Kor*, *Pdyn*, and *Tac2* genes in the arcuate and AVPV^Kiss1^ nuclei upon E2 treatment. **C** Expression of *Kiss1* and *Golt1a* in the arcuate and AVPV^Kiss1^ (*n* = 3-6). Error bars represent SEM. DeseqFDR **p < 0.01, ***p < 0.001, ns not significant. **D** ERα occupancy at the *Golt1a/Kiss1* locus in the arcuate and AVPV nuclei (http://genome-euro.ucsc.edu/). **E** ERE sequence detected for the *Greb1* (positive control), and the peaks identified proximal to *Kiss1*, *Pdyn*, and *Tac2*. Gene expression data were obtained from previously published studies^17^^,118^.

With the view of gaining further insight into ERα-mediated negative feedback in the arcuate nucleus, we compared arcuate and AVPV^KISS1^ transcriptional responses to estrogen. Specifically, we re-analysed previously published datasets from the Hrabovszky^17^ and Kauffman^118^ labs and asked whether genes affected by estrogen in the arcuate are similarly affected by estrogen in the AVPV^KISS1^, and whether these effects are also likely mediated directly by ERα-binding events. Overall, the proximity to an ERα site is associated with gene upregulation by estrogen treatment in both nuclei (Supplemental Fig. 1C). We also found that most of the genes affected by estrogen and containing proximal ERα peaks in the arcuate were similarly affected in the AVPV^KISS1^ neurons (Fig. 2A), demonstrating that region-specific modulation of gene expression by ERα is not a general feature of these nuclei.

Interestingly, our analysis of prior RNAseq datasets^17^^,118^ found that the *Kiss1 and Golt1a* genes were repressed in the arcuate nucleus and induced in AVPV^KISS1^-neurons (Fig. 2A–C), while being proximal to an arcuate-specific ERα binding site (Figs. 2D, E). The data analysis presented here highlights the fact that this pattern of activity is highly unusual, as no other genes with arcuate ERα binding sites displayed such striking opposing regulation (Fig. 2A). For example, other genes known to modulate *Kiss1*-neuron pulsatility^8,9,23^, the *Tac2* gene and the *Dynorphin* gene (*Pdyn*), were bound by ERα and regulated by estrogen only in the arcuate nucleus (Fig. 2A, B, Supplemental Fig. 1E, Supplemental Fig. 1D). *Golt1a* and *Kiss1* are expressed from a common genomic locus (*Kiss1-Golta1* locus)^24^, and evidence form our RNA-seq analysis support the proposition^24–27^ that they may be co-regulated by ERα (Fig. 2C). Importantly, the ERα binding site identified approximately 4 kb upstream of the *Kiss1* transcriptional start site was present in an intergenic region downstream of the *Golt1a* gene (Fig. 2D). These findings suggest that the *Kiss1-Golt1a* locus may be regulated by a proximal ERα binding site, and that a feature exclusive to the arcuate nucleus allows for ERα-driven repression in this locus. Overall, our analysis revealed region-specific ERα-bound genes that could modulate neuronal activity under negative feedback conditions, and a unique transcriptional control of *Kiss1* gene expression.

### *Dax1* is enriched in the arcuate hypothalamus and can repress *Kiss1*-transcription in vitro

**Fig. 3:**
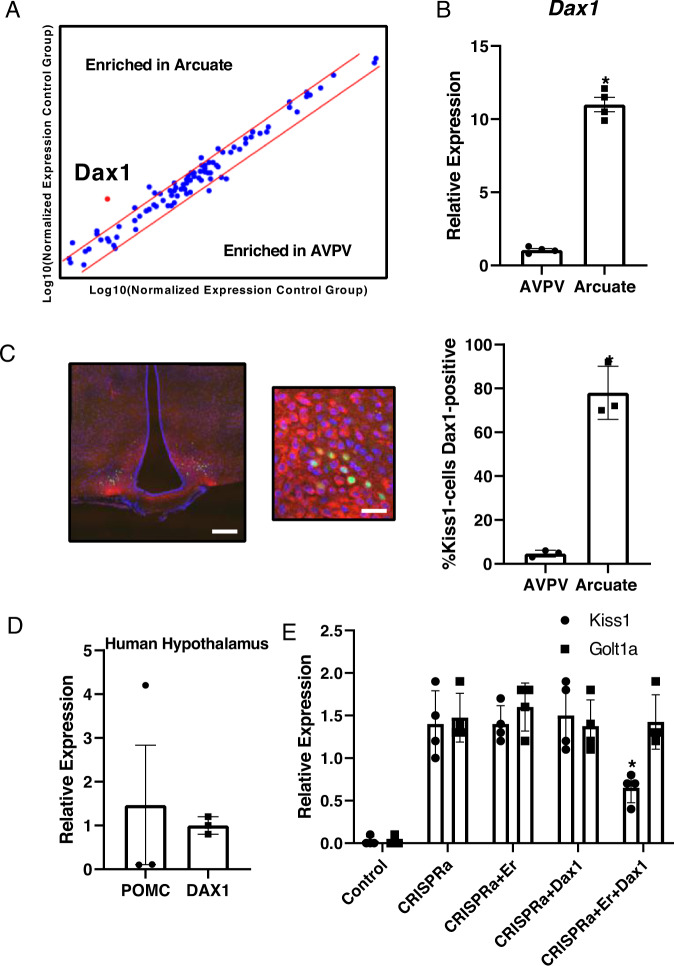
Dax1 is enriched in the arcuate hypothalamus and can repress *Kiss1*-transcription. **A** qPCR screening of 84 nuclear receptor co-regulators in the mouse arcuate and AVPV hypothalamus under estrogen stimulation (*n* = 4). **B** Confirmation of *Dax1* expression in the arcuate and AVPV hypothalamus using biological replicates under estrogen stimulation (*n* = 4) *p < 0.05 by t-test error bars represent SEM. **C** Detection of the DAX1 protein (red) by immunofluorescence in the whole arcuate and in *Kiss1*-neurons (green) in intact *Kiss1*-Cre-GFP mice in diestrous (*n* = 3) *p < 0.05 by t-test Error bars represent SEM. Scale bar 50 µm (left), 20 µm (right). **D** DAX1 and POMC in human hypothalamus (*n* = 3). Error bars represent SEM. **E** expression of *POMC* and *DAX1* in human arcuate nucleus (*n* = 3) Error bars represent SEM. **F** CRISPR-activation of the *Golt1a* promotor in C2C12 cells transfected with mERα or/and mDax1. Representative data from an experiment conducted twice (*n* = 4 wells per group) *p < 0.05 compared to *Golt1a* by Holm-Sidak multiple t-tests. Error bars represent SD. Source data are provided as a Source Data file.

The presence of nucleus-specific transcriptional co-regulators has been postulated as a possible mechanistic explanation for the opposing regulation of the *Kiss1* gene in the arcuate and AVPV nuclei^19^. In order to identify factors that may mediate region-specific ERα activity, we conducted a qPCR screen of 84 nuclear receptor co-regulators in the arcuate and AVPV under estrogen stimulation. Surprisingly, we found relatively few differentially-expressed genes (Fig. 3A). Of these, only one transcript, *Nr0b1* (also known as *Dax1*), was enriched in the arcuate nucleus by a factor of over 5-fold. We confirmed this result on biological replicates (Fig. 3B). We also analysed the same panel of co-regulators in the independently-generated RNA-seq data from arcuate and AVPV^KISS1^ nuclei^17,18^. *Dax1* was confirmed in these data to be the most highly enriched cofactor from our original panel of genes in the arcuate nucleus, and it was expressed at much lower levels in KISS1 neurons of the AVPV (Supplemental Fig. 3). Using immunohistochemistry, we found that many cells in the arcuate nucleus express the DAX1 protein (Fig. 3C). However, co-immunofluorescence experiments using animals that express GFP in *Kiss1* neurons revealed that at least 70% of *Kiss1* neurons in the arcuate nucleus express a detectable level of DAX1 protein (Fig. 3C). This compares to only approximately 5% of *Kiss1* neurons in the AVPV (Fig. 3C). We also detected *DAX1* expression by qPCR in the arcuate nucleus of postmenopausal women (Fig. 3D). Although it remains to be determined whether *DAX1* is present in *KISS1* neurons of humans, it is present in the hypothalamus at a level that is comparable to that of the known arcuate-expressed gene, *POMC* (Fig. 3D). Together, these studies identify DAX1 as a transcription factor that is selectively enriched in the arcuate hypothalamic nucleus compared to the AVPV, and is therefore a candidate-mediator of nucleus-specific estrogen action on *Kiss1*.

Dax1 is a nuclear receptor that is known to interact with ERα^28^, and serve as a transcriptional repressor in other steroid-responsive tissues^22,28^. As such, we asked whether DAX1 can inhibit *Kiss1*-expression under estrogen stimulation in vitro. In order to answer this question, we took advantage of the finding that the *Kiss1* gene can be regulated by the promotor of the upstream *Golt1a* gene^23^. This allowed us to use CRISPRa to activate *Golt1a* transcription, and then assess the effects of ERα and DAX1 on *Kiss1* mRNA transcription. As is conventional, we performed these experiments in cells that have very low endogenous transcription of the genes of interest, in this case muscle C2C12 cells cultured in 10 nM estrogen. We first confirmed that activation of the *Golt1a* promotor using CRISPRa resulted in increased expression of both the *Golt1a* gene and the *Kiss1*-gene (Fig. 3E). We then found that co-transfecting a Dax1 construct in combination with an ERα construct reduced *Kiss1*-expression compared to the empty-vector control (Fig. 3E). These data demonstrate that Dax1 can impede *Kiss1*-locus activation, likely from an ERα binding site identified in the *Golt1a*-*Kiss1* intergenic region (Fig. 2D).

### Mice lacking Dax1 in *Kiss1* neurons have abnormal *Kiss1* gene-regulation

**Fig. 4:**
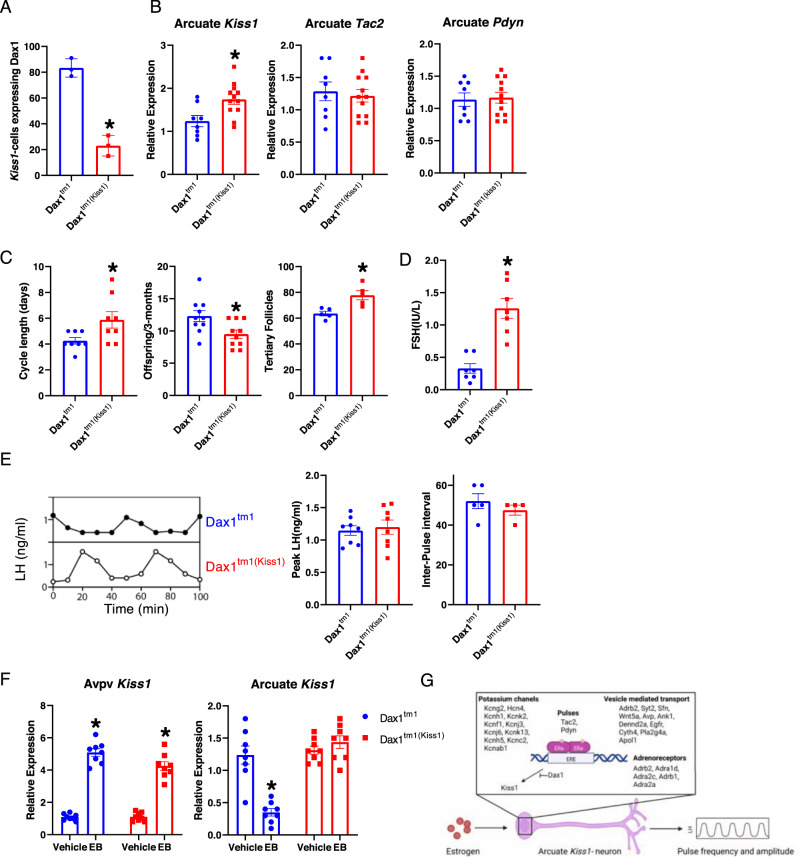
Mice lacking DAX1 in *Kiss1*-neurons have abnormal *Kiss1* gene regulation. **A** quantification of *Cre*-mediated deletion of DAX1-protein in arcuate *Kiss1*-neurons (*n* = 3 mice). **B** Expression of *Kiss1*, *Tac2*, and *Pdyn* in the arcuate nucleus of intact mice at 9am on the first day of diestrous (*n* = 8 Dax1^tm1^, *n* = 12 Dax1^th1(Kiss1)^ *p < 0.05. **C** Cycle length (*n* = 8), Offipring/3-months (*n* = 8 Dax1^tm1^, n = 10 Dax1^th1(Kiss1)^) *p < 0.05 by t-test, Tertiary follicles per ovary (*n* = 5). (**D**) Plasma FSH levels at 9am on the first day of diestrous (*n* = 7). **E** Representative LH profiles starting at 9am on the first day of diestrous. Peak LH (*n* = 8) an inter-pulse interval (*n* = 5 Dax1^tm1^, *n* = 4 Dax1^th1(Kiss1)^). **F** *Kiss1*-expression in the AVPV and arcuate following estrogen stimulation (*n* = 8) *p < 0.05 compared to vehicle 2-way ANOVA with Tukey’s multiple test correction. **G** Schematic representation of ERα function during negative feedback in arcuate *Kiss1*-neurons (Created with BioRender.com). Error bars represent SEM. Source data are provided as a Source Data file.

In order to determine whether Dax1 physiologically modulates *Kiss1* gene transcription, we generated mice lacking DAX1 in *Kiss1* cells. We crossed *Dax1*^*tm*^ mice with *Kiss1-cre* animals to produce *Dax1*^*tm1(kiss1)*^ mice and littermate controls (*Dax1*^*tm*^). This strategy resulted in the detection of DAX1 protein in only approximately 20% of Kiss1 neurons of *Dax1*^*tm1(kiss1)*^ mice compared to over 80% of *Dax1*^*tm1*^ mice (Fig. 4A). We found that intact female *Dax1*^*tm1(kiss1)*^ mice on the morning of diestrous had elevated *Kiss1* gene expression in the arcuate nucleus (Fig. 4B). Importantly, we found that the *Tac2* gene and the *Pdyn* gene were not differentially affected by deletion of *Dax1* in *Kiss1*-neurons of the arcuate nucleus (Fig. 4B), thus demonstrating the specificity of this transcriptional mechanism to the *Kiss1*-locus. *Dax1*^*tm1(kiss1)*^ mice displayed several subtle features that indicated the abnormal function of the HPO axis (Fig. 4C). However, despite having elevated plasma FSH levels (Fig. 4D), that likely still remained within the normal physiological range, we did not detect dramatic differences in LH-pulse dynamics in *Dax1*^*tm1(kiss1)*^ during diestrous (Fig. 4E). That being said, DAX1 in *Kiss1*-cells was required for exogenous estrogen-treatment mediated suppression of the *Kiss1*-gene in the arcuate nucleus (Fig. 4F). Therefore, the presence of DAX1 in arcuate kisspeptin-neurons specifically explains, at least in part, the unique opposing regulation of the *Kiss1* gene in the arcuate and AVPV nuclei of the hypothalamus. Furthermore, the DAX1-dependent restraint of *Kiss1* transcription in the arcuate essentially couples it to the activity of the neuron, and loss of this restraint results in elevated FSH secretion and an ovarian hyperstimulation syndrome.

Together, our analyses demonstrate that arcuate-specific processes, such as modulation of pulsatility, may be defined by region-specific ERα binding events (Fig. 1 and Fig. 2). In addition, while DAX1 in *Kiss1* cells does not appear crucial for the maintenance of episodic gonadotropin release, it does provide mechanistic insight into the unique control of *Kiss1* gene-transcription, and how it may be coupled to the activity of the neuron (Fig. 3 and Fig. 4). Taken together, these analyses provide an ERα-centric model (Fig. 4G) to explain estrogen action on *Kiss1* neurons of the arcuate hypothalamic nucleus.

## Discussion

We have investigated the mechanistic basis for hypothalamic estrogen sensing, with a focus on negative feedback in the arcuate hypothalamic nucleus. Our findings have revealed a complex network of genes that are bound by ERα and regulate neurotransmitter processing and release pathways in an estrogen-responsive way. We found that the intersect between arcuate and AVPV peaks was considerably more similar than a comparison of either nucleus to ERα binding events observed in mouse breast tissue^28^ (Supplementary Figure 1B). Therefore, our initial analyses also demonstrate that ERα interacts with the genome in hypothalamic nuclei in a functionally neuron-specific manner. In addition, we identified ERα binding events in the proximity of key *Kiss1*-neuron functional genes in the arcuate nucleus; *Tac2* and *Pdyn*, and the *Kiss1* gene itself. Indeed, we conducted mechanistic studies on *Kiss1* due to its unique pattern of expression and found that a nuclear receptor co-repressor, DAX1, is required for full ERα-dependent estrogen-negative feedback on this gene in the arcuate nucleus.

The recent use of optogenetics and fibre photometry have revolutionised our understanding of the kisspeptin system^24^. These techniques have firmly established that abrupt episodes of arcuate nucleus kisspeptin-neuron activity are responsible for determining gonadotropin pulsatility^7,25^. Under physiological conditions, the frequency of these arcuate episodes are under constant restraint by estrogen^20^. Therefore, arcuate *Kiss1* neurons must possess mechanisms to sustain neurotransmitter release, as well as modulate neuronal activity. Our data suggest that ERα plays a role in both of these processes. It appears to couple *Kiss1* transcription to metabolic need, reducing its production in the arcuate, via DAX1, as estradiol levels rise. It also controls pathways for the processing and secretion of neuropeptides, and likely modulates the neuronal pulsatility in the arcuate at least in part by binding to regulatory elements that we identified proximal to both *Tac2* and *Pdyn*. The focus of our studies has been negative feedback in the arcuate nucleus, which requires intricate control in order to maintain episodic gonadotropin pulses. However, ERα also likely plays a major role in coupling *Kiss1* transcription and neurotransmitter processing pathways to physiological positive feedback in the AVPV nucleus, a process that triggers the LH-surge and also requires interaction with circadian circuits^10^.

Functional insight into our ChIP-seq data was made possible through a comparative analysis of independently generated transcriptomic data from arcuate^17^ and AVPV^KISS1^-neurons^18^. Many estrogen-responsive genes contained proximal ERα binding sites (Supplemental files 14 and 5). Those which did not are potentially directly regulated by ERα via distal enhancers^29^, indirectly via other ERα-induced transcription factors, or via non-genomic actions of ERα (discussed below). Some gene regulation events, such as repression of *Tac2* and *Pdyn* may be explained simply by region-specific developmental chromatin architecture at ERα-binding-sites. However, explaining opposing gene expression, such as that required of *Kiss1* in the arcuate and AVPV is more challenging. Indeed, our comparative analyses suggest that the regulation of the *Kiss1* locus may, in fact, be unique in these hypothalamic nuclei. We found that the known ERα co-repressor DAX1 explains, at least in part, estrogen-mediated *Kiss1* repression in the arcuate nucleus. This did not result in dramatic differences in LH pulsatility in mice lacking DAX1 in *Kiss1* cells, which is broadly consistent with the maintenance of *Pdyn* and *Tac2* expression. However, we did not perform fibre photometry to directly measure kiss1 neuron activity. Therefore, there may have been subtle changes in LH dynamics that were not detected by our sampling paradigm. It is also formally possible that *Dax1* deletion in other *Kiss1*-cells contributes to the subtle reproductive phenotype of ovarian hyperstimulation syndrome. We also note here the unusual structure of the *Kiss1* locus^23^, the presence of an arcuate-specific enhancer (−12 890 to −2165 bp relative to *Kiss1* TSS)^26^ that overlaps with the ERα-binding site that we identified, its transcriptional relationship with the upstream *Golt1a* gene^27,30,31^, and the fact that it has been found to be an anchor-point for long-range ERα-bound DNA loops^32^. Together, these findings highlight the highly complex nature of *Kiss1*-transcription, a process that has necessarily evolved as part of the ‘two-nucleus’ hypothalamic mechanism that sustains fertility by controlling follicular development via the arcuate^8^, and ovulation via the AVPV^10^.

Our experiments have focused on so called ‘classical’ ERα-signalling. That is, direct binding of the receptor to estrogen response elements (EREs) in the genome to regulate gene transcription. However, two previous reports concluded that ‘non-classical’, ERE-independent, signalling is partially responsible for mediating estrogen negative feedback^29,33^. The interpretation of findings in these mice is complicated by the fact that the mutated ERα can still bind ligand and therefore may physically interact with, and modify the function of, other transcription factors (possibly including DAX1) in a way that would not necessarily occur if it could bind to the genome in the usual way. Nevertheless, it remains likely that both classical and non-classical estrogen signalling are required for the normal hypothalamic control of fertility. Finally, we acknowledge the presence of species differences in the kisspeptin system^34,35^, and that the role of ERα and DAX1 in the control of negative feedback in humans remains to be determined.

Previous findings in the literature had seemed to contradict an ERα-centric hypothesis for negative feedback^36–42^. However, during the review of this manuscript, ERα was conclusively shown by others to be critical for estrogen-negative feedback on *Kiss1*-neuron pulsatility in the arcuate hypothalamic nucleus^43^. Our data provide a potential transcriptional framework for this single-receptor phenomenon, and identify DAX1 as an important regulator of the highly complex *Kiss1*-locus in the arcuate hypothalamic nucleus.

## Methods

### In vivo experiments

All procedures were conducted on female mice and approved under the U.K Animals (Scientific Procedures) Act, 1986, and approved by the Animal Welfare Ethical Review Body of Imperial College London. Animals were housed under standard conditions in individually ventilated cages with free access to food and water, environmental enrichment, and wood-chip bedding. Lights-on 07:00 h, lights of 19:00 h. *Dax1*^*tm1*^ ((B6Ei.129-*Nr0b1*^*tm1Lja*^/EiJ) Stock number 007006) and *Kiss1-Cre* ((Kiss1^tm1.1(cre/EGFP)Stei^) Stock number 017701) mice were obtained from The Jackson Laboratory. *Kiss1-Cre* were maintained as heterozygous animals with the exception of homozygous used for co-immunohistochemistry. A survey of steroidogenic gene expression was conducted in *Dax1*^*tm1(Kiss1)*^ and did not identify any abnormalities in the ovary or adrenal gland. However, we cannot exclude the formal possibility that the deletion of *Dax1* in extra-hypothalamic *Kiss1*-cells may confound the interpretation of our results. Assessment of the estrous cycle was performed by vaginal cytology. Tissues from animals is diestrous were collected at 9 am. Animals were typically sacrificed between 8 and 12 weeks old.

### Estrogen-treatment

Animals were ovariectomised and implanted with 2 cm sub-cutaneous Silastic tubes (Dow Corning 508-005) containing 17β-estradiol (Merk) in sesame oil (0.1 mg/mL). One week later, they were injected at 09:00 with either vehicle (sesame oil) or estradiol benzoate (0.05 mg/kg), and tissues were collected 28 h after the injection.

### Human samples

Anonymised human samples were obtained from the Cambridge Brain Bank (CBB) with informed consent under CBB license (NRES 10/HO308/56) approved by the East of England—Cambridge Central Research Ethics Committee. Subjects were approached in life for written consent for brain banking, and all tissue donations were collected and stored following legal and ethical guidelines (NHS reference number 11/0EE/0011). All three donors were female, the age at death ranged from 74-85, and the postmortem interval ranged from 27-46 h.

*ChIP-Seq* was performed by Active Motif using 25ug of pooled chromatin from arcuate and AVPV tissue dissected from C57BL/6 mice and 40ul of antibody (sc-543), with four replicates per condition. 75-nt single-end sequence reads were generated by Illumina sequencing (NextSeq 500) and aligned to the mouse reference genome mm10 using bowtie2 (version 2.5.0)^29^. Enriched regions were identified by using MACS2^34^. Coverage tracks were generated with DeepTools 3.5^35^ bamCoverage with options “--binsize 50 --normalizeUsing CPM --effectiveGenomeSize 2652783500 --extendReads 300.” Input tracks were thereafter subtracted by using bigwigCompare. For the heatmap display, computeMatrix was used on regions defined by MACS separated by arcuate or AVPV enrichment, with options “--referencePoint center -a 2000 -b 2000 -bs 50.” Estrogen Response Elements (ERE) and genomic annotations were defined using HOMER annotatepeaks^36^. HOMER annotatepeaks was also used to define genes proximal to ERα peaks, and to perform Gene Ontology term analysis against the Reactome database^20^. Images of ERα occupancy at individual loci were generated with the UCSC genome browser^14^. ChIP-seq data for ERα in mouse breast tissue (GSE130032)^44^ was accessed through the Gene Expression Omnibus^37^. Raw reads were processed according to the pipeline described for our ERα ChIP-seq data for the mouse genome annotation mm10.

*RNA-Seq* data from two previously published reports on the mouse arcuate and KISS1 neurons from the arcuate and the AVPV nuclei were accessed through the Sequence Read Archive, with bioproject numbers PRJNA686688^17^ and PRJNA706198^18^. Raw reads were aligned to the mouse reference genome mm10 using HISAT2^45^ and associated to ENSEMBL transcript annotation GRCm38.102 using feature-Counts from the Rsubread package (version 2.10.5)^46^. Differential expressed (DE) genes upon E2 treatment were determined with DESeq2 package (version 1.36.0)^47^. Shrinkage of effect size was performed on Deseq2 results using the *apeglm* method through the function lfcShrink^48^. Expression heatmaps were generated with gplots (ver 3.1.3) heatmap.2 using the log2 transformation of transcripts per million (TPM) and scaling per gene (Row). Rsubread, Deseq2 and lfcShrink are Bioconductor packages (Release 3.15) and were executed in RStudio 2022.07.1 Build 554 under R 4.2.0. Distance from gene loci to ERα peaks was determined using Bedtools closestbed^49^ and values lower than 20 kBp were considered proximal. As a quality control, sequencing coverage was computed for every dataset using DeepTools 3.5^35^ plotCoverage with options “--region X --BED GRCm38.102.bed”. Sequencing coverage for RNAseq datasets from isolated arcuate Kiss1-neurons precluded direct comparison to AVPV^KISS1^ neurons datasets (Supplemental Fig. 4, Supplemental Data 7).

### Gene expression analyses

cDNA (Invitrogen VILO cDNA Synthesis Kit) was prepared from biopsies of mouse tissue guided by the mouse brain atlas. Nuclear receptor co-regulators were screened by RT2 Profiler qPCR Array (Qiagen). Individual gene expression was quantified using the SYBR Green (Bio-Rad) method, using commercially available primers (Sigma Aldrich) and analysed by the ddCT-method. For human tissue samples, RNA was extracted using Qiagen RNeasy Universal Mini Kit with DNase1 digestion following manufacturer’s instructions. The RNA was then used to generate to cDNA via MMLV reverse transcriptase (Promega). qPCR was performed on human samples using TaqMan probes specific for *NR0B1* (Hs05033649_g1), *POMC* (Hs01596743_m1) and *GAPDH* (Hs02786624_g1) and Taqman Universal PCR Master Mix (ThermoFisher) on an Applied Biosystems Quantstudio 7 qPCR instrument (ThermoFisher). The PCR conditions are as follows: 50^o^C for 2 min, 95^o^C for 10 min, then 40 cycles of 95^o^C for 15 s and 60^o^C for 1 min.

### Immunohistochemistry and immunofluorescence

Animals underwent perfusion fixation using 4% formaldehyde under terminal anaesthesia. Brains were further fixed at 4 ^o^C over-night, and then cryo-preserved in 30% sucrose. 40 µm sections were prepared using a sledge microtome. Samples were incubated with Immunostaining permeabilisation buffer (0.5% Triton X-100 in PBS 1X) for 30 min at 4 °C, then Immunostaining blocking buffer (0.3% Triton X-100 in PBS 1X with 10% Normal Donkey Serum) at room temperature for 1 h. Samples were incubated with the primary antibody in Immunostaining blocking buffer (0.3% Triton X-100 in PBS 1X with 10% Normal Donkey Serum) overnight at 4 °C. Primary antibodies were used at a concentration of 1:200 for the Anti-DAX1, clone 2F4 antibody (Merck, MABD398), and 1:250 for GFP Polyclonal Antibody, Alexa Fluor 488 (ThermoFisher Scientific, A-21311). Samples were incubated with secondary antibody (Strateck 715-165-151-JIR) 1:1000 for at least 2 h at room temperature. Samples were then stained with DAPI (ThermoFisher Scientific, D1306) at a concentration of 1:1000 diluted. Slides were dried and mounted with VECTASHIELD Mounting Medium for Fluorescence (Vector Laboratories, H-1000). Slides were imaged using a Zeiss LSM-780 Inverted Confocal Microscope (Zeiss) and analysed using ImageJ software.

*CRISPRa* was performed using Edit-R reagents from Dharmacon in C2C12 cells obtained from the American Type Culture Collection. Briefly, cells were cultured in media containing 10 nM 17β-estradiol (Merck), and transfected (DharmaFECT kb DNA Transfection Reagent) with a set of four lentiviral sgRNA constructs (GSGM 11893-247351096-247351090) targeted to the Golt1a promotor (or a non-targeting control (GSGC1193), and a lentiviral mCMV-Blast-dCas9-VPR plasmid (CAS11915). Transcripts were quantified by gene expression analysis. Expression constructs for DAX1 and ERα were purchased from Active Motif.

### Hormone measurement

LH was measured as previously described^50^. Animals were acclimatised to handling for two weeks. 6ul of plasma was collected every 10 min, starting at approximately 9am on the first day of diestrous. The 25%-change threshold method was used to identify peaks, which has been shown to provide accurate results in intact animals^51^. Peak LH was defined as the maximal LH level of a pulse, and averaged for animals that underwent multiple pulses during the sampling period. Inter-peak interval was measured for individual animals that underwent more than one pulse during the sampling period. Mouse Follicle Stimulating Hormone was measured according to the manufacturer’s instruction (sensitivity of assay 0.1 ng/ml MyBioSource, MBS727159).

### Statistical analysis

See the relevant section above for description of ChIP-seq and RNA-seq statistical analysis. All other analyses were conducted using GraphPad Prism 8.2.1. Man Whitney U tests were used to compare means between two experimental groups. Two-tailed Two-way ANOVA followed by Sidak multiple comparison correction was used to compare multiple groups.

## Supplementary information

Supplementary Information

Description of Additional Supplementary Files

Supplementary Data 1

Supplementary Data 2

Supplementary Data 3

Supplementary Data 4

Supplementary Data 5

Supplementary Data 6

Supplementray Data 7

## Data Availability

ChIP-seq data generated in this study are publicly available at the Gene Expression Omnibus^37^ GSE227540. Publicly available data re-analysed in this study were obtained as follows: Arcuate RNA-seq data18 accession PRJNA686688, AVPVKISS1 RNA-seq data17 accession number PRJNA706198, Breast ChIP-seq data22 accession number GSE130032. All other data supporting the findings of this study are available within the paper and its Supplementary Information. Source data are provided with this paper.

## Source data

Source Data
