# Supplementary material for: Dax1 modulates ERα-dependent hypothalamic estrogen sensing in female mice
Source: Nat Commun. 2023 May 29;14:3076. doi: 10.1038/s41467-023-38618-y (PMC10227040; doi:10.1038/s41467-023-38618-y)
Supplement: Supplementary file 1 — Supplementary Information [file 41467_2023_38618_MOESM1_ESM.pdf]

Supplemental figure 1

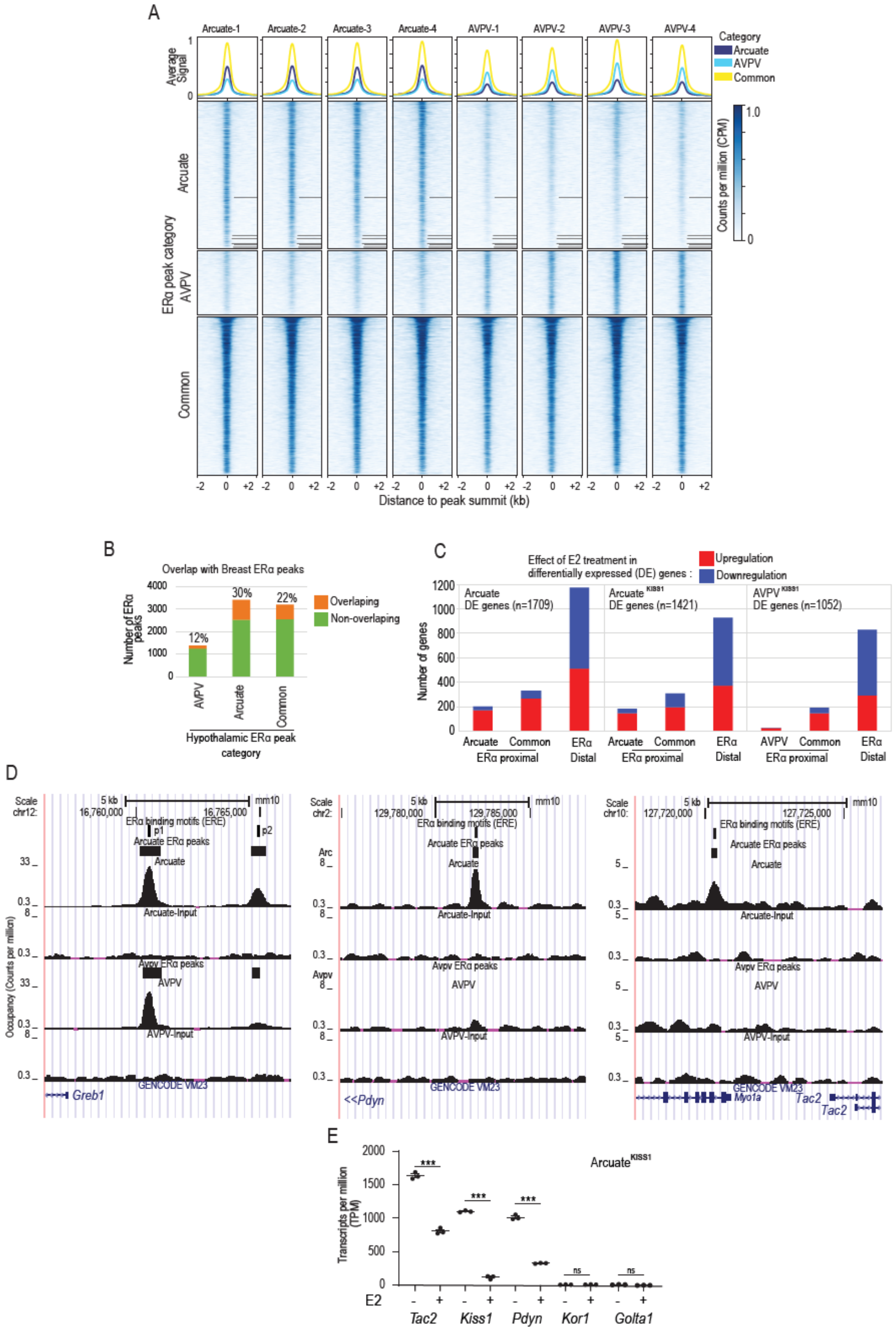

Supplementary Figure 1. ERα ChIP-Seq in female Arcuate and AVPV nucleus. (A) Profile plots and heatmaps of ERα occupancy in independent ChIP-seq samples. (B) Number of hypothalamus ERα peaks overlapping with breast ERα peaks, separated by nucleus enrichment. (C) Transcriptional effect of estrogen on ERα-proximal genes. (D) ERα occupancy at peaks proximal to the *Greb1, Pdyn, and Tac2* loci in the arcuate and AVPV nuclei. Error bars represent SEM. DeseqFDR **p<0.01, ***p<0.001, ns not significant. (E) Gene expression of *Tac2, Kiss1, Pdyn, Kor1* and *Golta1* in isolated *Kiss1*-positive arcuate neurons upon E2 treatment (n=3). Data were compared using 1-way

ANOVA/Sidak. $***$ $p<0.001$, ns not significant. Gene expression data from previously published studies[17,118]

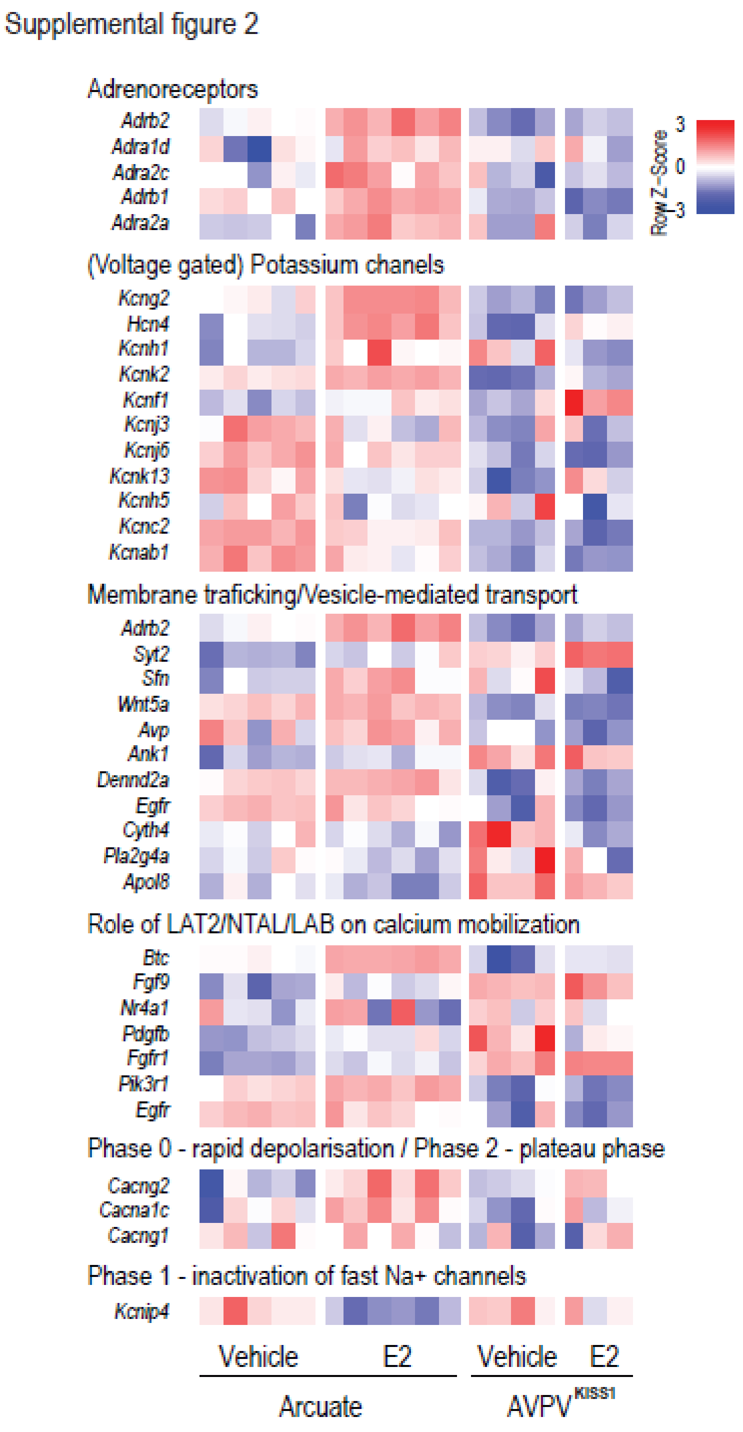

Supplementary Figure 2. Expression of candidate genes from enriched GO terms from panel A in the arcuate and AVPV nuclei upon E2 treatment (RNAseq). Gene expression data from previously published studies[17,118]

Supplemental figure 3

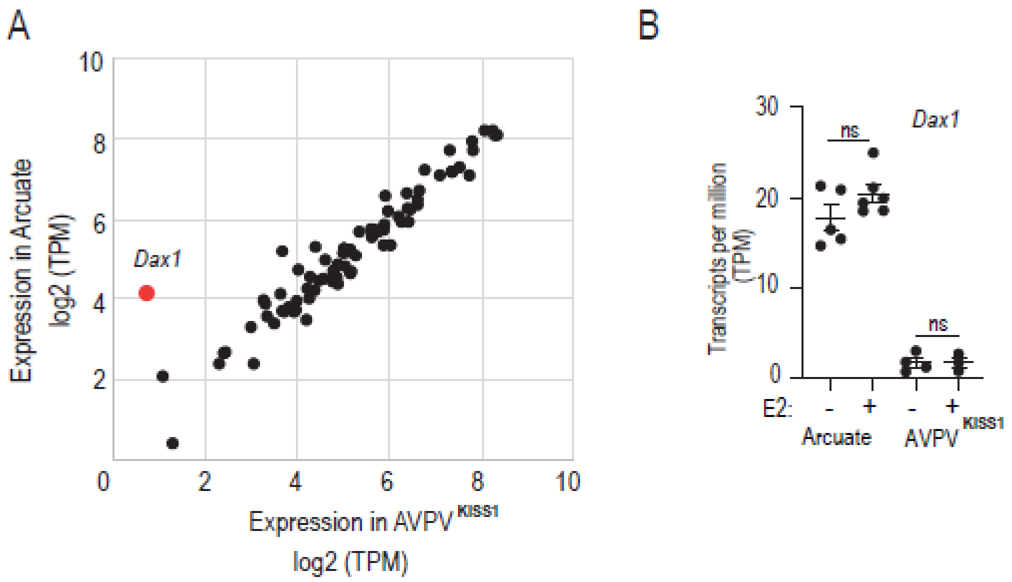

Supplementary Figure 3. Nuclear receptor and co-regulator expression in RNA-seq data (A) RNA-seq expression of 84 nuclear receptor co-regulators in the mouse arcuate and AVPV hypothalamus under estrogen stimulation. (B) Expression of Dax1 in the arcuate and AVPV nuclei upon E2 treatment (n=3-6). Data were compared using 2-way ANOVA/Sidak. ns not significant. Gene expression data from previously published studies[17,118]

Supplemental figure 4

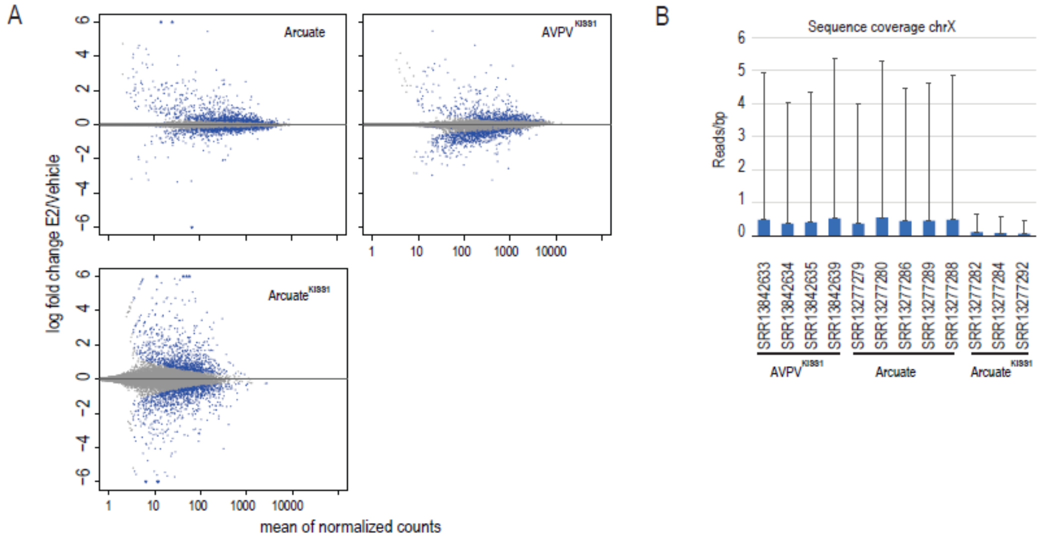

Supplementary Figure 4. MAplots and sequence coverage of RNA-Seq data.(A) MAplots upon E2 treatment from arcuate and isolated Kiss1 positive arcuate and AVPV neurons (RNAseq). (B) Sequencing coverage of gene loci at the X chromosome in “Vehicle” samples (n=3-6). Error bars represent SEM. Gene expression data from previously published studies[17,118]
