# Supplementary material for: Dax1 modulates ERα-dependent hypothalamic estrogen sensing in female mice
Source: Nat Commun. 2023 May 29;14:3076. doi: 10.1038/s41467-023-38618-y (PMC10227040; doi:10.1038/s41467-023-38618-y)
Supplement: Supplementary file 2 — Description of Additional Supplementary Files [file 41467_2023_38618_MOESM2_ESM.pdf]

Description of Additional Supplementary Files

File Name: Supplementary Data 1
Description: ERα peaks in Arcuate and AVPV, total or separated according to differential enrichment, ERα peaks in mammary gland, and Dax1 peaks in ES cells.

File Name: Supplementary Data 2
Description: *De novo motif analysis in peaks from* ERα and Dax1 *ChIP-seq datasets.*

File Name: Supplementary Data 3
Description: *Quantification of gene expression in RNA-seq samples, expressed as transcripts per million.*

File Name: Supplementary Data 4
Description: *Distance calculation between all* Arcuate or AVPV ERα peaks and *genes included in the* GRCm38.102 annotation.

File Name: Supplementary Data 5
Description: Differentially expressed genes after E2 treatment in RNA-seq datasets.

File Name: Supplementary Data 6
Description: GO term analysis of ChIP-seq peaks against the Reactome database.

File Name: Supplementary Data 7
Description: Metadata and QC
