# Supplementary material for: Dax1 modulates ERα-dependent hypothalamic estrogen sensing in female mice
Source: Nat Commun. 2023 May 29;14:3076. doi: 10.1038/s41467-023-38618-y (PMC10227040; doi:10.1038/s41467-023-38618-y)
Supplement: Supplementary file 4 — Supplementary Data 2 [file 41467_2023_38618_MOESM4_ESM.pdf]

Homer *de novo* Motif Results (./MotifSearch/homer/Arcuate-all/)

Known Motif Enrichment Results

Gene Ontology Enrichment Results

If Homer is having trouble matching a motif to a known motif, try copy/pasting the matrix file into STAMP
More information on motif finding results: HOMER | Description of Results | Tips
Total target sequences = 6612

Total background sequences = 42577
* - possible false positive

Rank	Motif	P-value	log P-pvalue	% of Targets	% of Background	STD(Bg STD)	Best Match/Details	Motif File
1	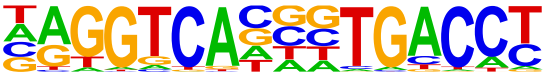	1e-2669	-6.147e+03	31.26%	0.71%	40.6bp (57.8bp)	ERE(NR),IR3/MCF7-ERa-ChIP-Seq(Unpublished)/Homer(0.977) More Information | Similar Motifs Found	motif file (matrix)
2	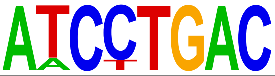	1e-247	-5.705e+02	13.28%	3.46%	51.2bp (66.1bp)	SIX1/MA1118.1/Jaspar(0.712) More Information | Similar Motifs Found	motif file (matrix)
3	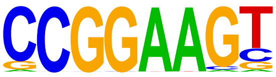	1e-163	-3.767e+02	11.39%	3.61%	55.1bp (59.7bp)	ELF1(ETS)/Jurkat-ELF1-ChIP-Seq(SRA014231)/Homer(0.983) More Information | Similar Motifs Found	motif file (matrix)
4	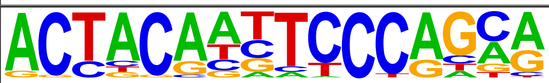	1e-120	-2.777e+02	3.25%	0.38%	50.9bp (57.4bp)	THAP11/MA1573.1/Jaspar(0.948) More Information | Similar Motifs Found	motif file (matrix)
5	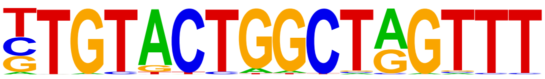	1e-103	-2.390e+02	2.04%	0.14%	39.8bp (43.6bp)	FOXA1:AR(Forkhead,NR)/LNCAP-AR-ChIP-Seq(GSE27824)/Homer(0.632) More Information | Similar Motifs Found	motif file (matrix)
6	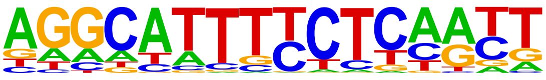	1e-84	-1.945e+02	2.00%	0.19%	59.8bp (56.6bp)	PU.1(ETS)/ThioMac-PU.1-ChIP-Seq(GSE21512)/Homer(0.642) More Information | Similar Motifs Found	motif file (matrix)
7	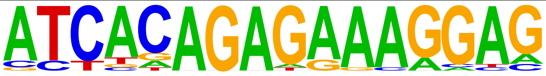	1e-77	-1.784e+02	1.51%	0.10%	47.6bp (51.2bp)	PRDM1/MA0508.3/Jaspar(0.720) More Information | Similar Motifs Found	motif file (matrix)
8	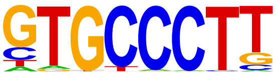	1e-63	-1.455e+02	25.06%	16.87%	53.1bp (59.2bp)	ERRg(NR)/Kidney-ESRRG-ChIP-Seq(GSE104905)/Homer(0.842) More Information | Similar Motifs Found	motif file (matrix)
9	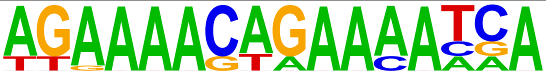	1e-59	-1.359e+02	0.47%	0.00%	50.3bp (10.0bp)	Stat2/MA1623.1/Jaspar(0.759) More Information | Similar Motifs Found	motif file (matrix)
10	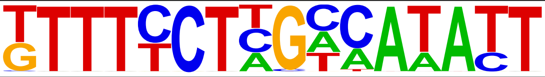	1e-52	-1.201e+02	0.42%	0.00%	56.2bp (0.0bp)	PB0006.1_Bcl6b_1/Jaspar(0.610) More Information | Similar Motifs Found	motif file (matrix)
11	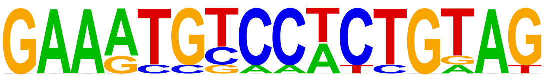	1e-49	-1.149e+02	0.51%	0.01%	55.9bp (10.6bp)	ZNF528(Zf)/HEK293-ZNF528.GFP-ChIP-Seq(GSE58341)/Homer(0.593) More Information | Similar Motifs Found	motif file (matrix)
12	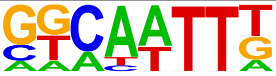	1e-47	-1.101e+02	24.17%	17.08%	57.1bp (63.4bp)	PH0024.1_Dlx5/Jaspar(0.750) More Information | Similar Motifs Found	motif file (matrix)
13	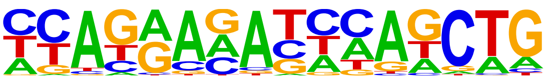	1e-46	-1.079e+02	2.78%	0.77%	56.4bp (60.4bp)	Ap4(bHLH)/AML-Tfap4-ChIP-Seq(GSE45738)/Homer(0.590) More Information | Similar Motifs Found	motif file (matrix)
14	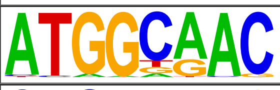	1e-45	-1.057e+02	7.70%	3.87%	55.7bp (61.4bp)	RFX3/MA0798.2/Jaspar(0.831) More Information | Similar Motifs Found	motif file (matrix)
15	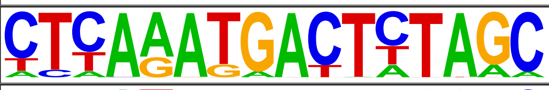	1e-45	-1.054e+02	0.44%	0.00%	48.6bp (56.1bp)	USF1/MA0093.3/Jaspar(0.551) More Information | Similar Motifs Found	motif file (matrix)
16	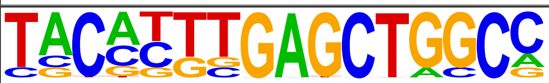	1e-45	-1.046e+02	0.38%	0.00%	42.8bp (5.4bp)	ZBTB6/MA1581.1/Jaspar(0.573) More Information | Similar Motifs Found	motif file (matrix)
17	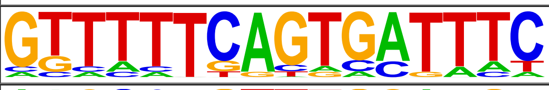	1e-43	-1.008e+02	0.42%	0.01%	52.4bp (0.0bp)	GFI1/MA0038.2/Jaspar(0.636) More Information | Similar Motifs Found	motif file (matrix)
18	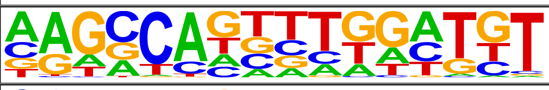	1e-43	-9.953e+01	0.36%	0.00%	38.1bp (0.0bp)	ZFP42/MA1651.1/Jaspar(0.594) More Information | Similar Motifs Found	motif file (matrix)
19	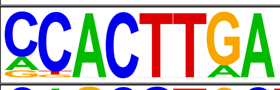	1e-42	-9.842e+01	4.78%	1.99%	53.9bp (61.2bp)	NKX2-3/MA0672.1/Jaspar(0.949) More Information | Similar Motifs Found	motif file (matrix)
20	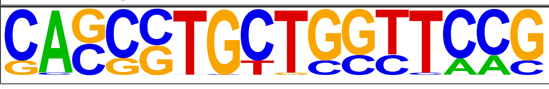	1e-37	-8.691e+01	1.21%	0.18%	51.2bp (63.1bp)	Zic1::Zic2/MA1628.1/Jaspar(0.630) More Information | Similar Motifs Found	motif file (matrix)

21	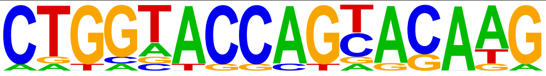	1e-36	-8.455e+01	0.32%	0.00%	41.3bp (41.8bp)	ZBTB12/MA1649.1/Jaspar(0.552) More Information | Similar Motifs Found	motif file (matrix)
22	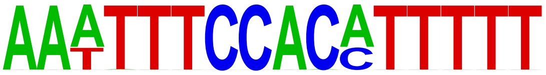	1e-36	-8.305e+01	0.36%	0.01%	55.3bp (0.0bp)	NFAT5/MA0606.1/Jaspar(0.631) More Information | Similar Motifs Found	motif file (matrix)
23	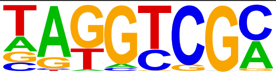	1e-35	-8.281e+01	8.14%	4.56%	52.6bp (66.9bp)	ZNF135/MA1587.1/Jaspar(0.673) More Information | Similar Motifs Found	motif file (matrix)
24	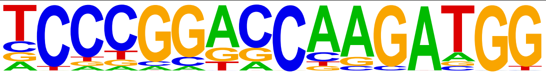	1e-34	-7.923e+01	1.35%	0.26%	58.0bp (61.6bp)	YY1/MA0095.2/Jaspar(0.662) More Information | Similar Motifs Found	motif file (matrix)
25	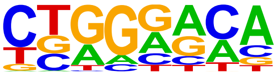	1e-32	-7.501e+01	9.80%	6.00%	52.4bp (59.1bp)	AR-halfsite(NR)/LNCaP-AR-ChIP-Seq(GSE27824)/Homer(0.768) More Information | Similar Motifs Found	motif file (matrix)
26	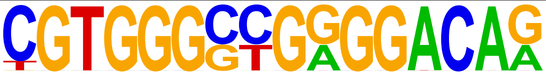	1e-31	-7.148e+01	1.21%	0.23%	54.3bp (66.1bp)	PB0107.1_Ascl2_2/Jaspar(0.632) More Information | Similar Motifs Found	motif file (matrix)
27	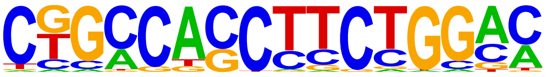	1e-31	-7.139e+01	0.97%	0.14%	54.3bp (67.4bp)	CTCF(Zf)/CD4+-CTCF-ChIP-Seq(Barski_et_al.)/Homer(0.696) More Information | Similar Motifs Found	motif file (matrix)
28	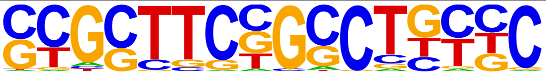	1e-28	-6.643e+01	2.00%	0.62%	53.3bp (65.4bp)	Zfx/MA0146.2/Jaspar(0.623) More Information | Similar Motifs Found	motif file (matrix)
29	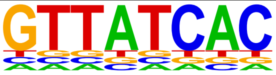	1e-20	-4.762e+01	1.15%	0.31%	52.3bp (56.9bp)	GATA5/MA0766.2/Jaspar(0.813) More Information | Similar Motifs Found	motif file (matrix)
30	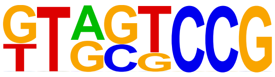	1e-20	-4.677e+01	5.49%	3.26%	56.6bp (65.6bp)	HINFP(Zf)/K562-HINFP.eGFP-ChIP-Seq(Encode)/Homer(0.684) More Information | Similar Motifs Found	motif file (matrix)
31	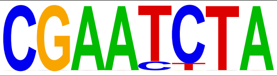	1e-15	-3.579e+01	1.36%	0.50%	56.8bp (67.1bp)	GATA1/MA0035.4/Jaspar(0.716) More Information | Similar Motifs Found	motif file (matrix)
32	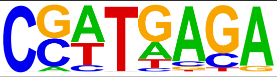	1e-14	-3.250e+01	1.95%	0.91%	60.3bp (60.9bp)	BARHL1/MA0877.2/Jaspar(0.696) More Information | Similar Motifs Found	motif file (matrix)
33 *	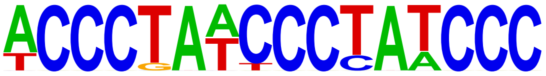	1e-6	-1.573e+01	0.14%	0.01%	51.2bp (62.4bp)	PB0107.1_Ascl2_2/Jaspar(0.555) More Information | Similar Motifs Found	motif file (matrix)
34 *	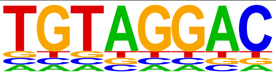	1e-4	-1.076e+01	0.82%	0.44%	49.7bp (59.8bp)	PH0065.1_Hoxc10/Jaspar(0.651) More Information | Similar Motifs Found	motif file (matrix)
35 *	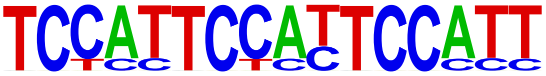	1e-3	-8.081e+00	0.06%	0.01%	44.1bp (4.9bp)	ZNF189(Zf)/HEK293-ZNF189.GFP-ChIP-Seq(GSE58341)/Homer(0.616) More Information | Similar Motifs Found	motif file (matrix)

Homer *de novo* Motif Results (./MotifSearch/homer/AVPV-all/)

Known Motif Enrichment Results

Gene Ontology Enrichment Results

If Homer is having trouble matching a motif to a known motif, try copy/pasting the matrix file into STAMP

More information on motif finding results: HOMER | Description of Results | Tips

Total target sequences = 4739

Total background sequences = 43000

* - possible false positive

Rank	Motif	P-value	log P-pvalue	% of Targets	% of Background	STD(Bg STD)	Best Match/Details	Motif File
1	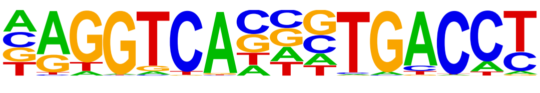	1e-2032	-4.680e+03	33.38%	0.79%	41.0bp (58.6bp)	ERE(NR),IR3/MCF7-ERa-ChIP-Seq(Unpublished)/Homer(0.977) More Information | Similar Motifs Found	motif file (matrix
2	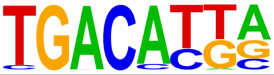	1e-214	-4.934e+02	40.59%	20.58%	51.4bp (67.4bp)	MEIS1/MA0498.2/Jaspar(0.883) More Information | Similar Motifs Found	motif file (matrix
3	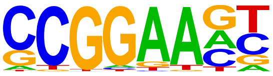	1e-161	-3.715e+02	16.58%	5.57%	54.1bp (64.6bp)	Elk4(ETS)/Hela-Elk4-ChIP-Seq(GSE31477)/Homer(0.979) More Information | Similar Motifs Found	motif file (matrix
4	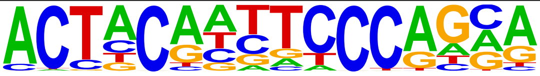	1e-159	-3.683e+02	5.49%	0.56%	52.2bp (59.3bp)	THAP11/MA1573.1/Jaspar(0.935) More Information | Similar Motifs Found	motif file (matrix
5	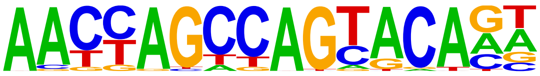	1e-85	-1.960e+02	2.17%	0.13%	46.1bp (44.5bp)	FOXA1:AR(Forkhead,NR)/LNCAP-AR-ChIP-Seq(GSE27824)/Homer(0.586) More Information | Similar Motifs Found	motif file (matrix
6	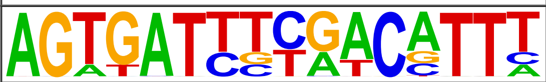	1e-68	-1.582e+02	0.70%	0.00%	51.9bp (0.0bp)	PH0037.1_Hdx/Jaspar(0.631) More Information | Similar Motifs Found	motif file (matrix
7	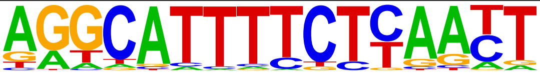	1e-67	-1.555e+02	2.30%	0.23%	61.4bp (53.4bp)	PB0148.1_Mtf1_2/Jaspar(0.579) More Information | Similar Motifs Found	motif file (matrix
8	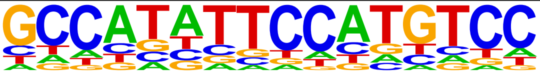	1e-61	-1.412e+02	0.63%	0.00%	53.2bp (0.0bp)	NFATC4/MA1525.1/Jaspar(0.604) More Information | Similar Motifs Found	motif file (matrix
9	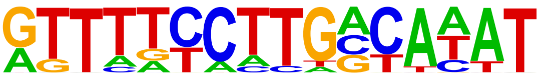	1e-61	-1.409e+02	0.78%	0.01%	53.5bp (31.1bp)	NF1:FOXA1(CTF,Forkhead)/LNCAP-FOXA1-ChIP-Seq(GSE27824)/Homer(0.654) More Information | Similar Motifs Found	motif file (matrix
10	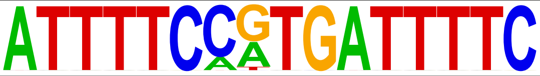	1e-58	-1.354e+02	0.70%	0.01%	48.0bp (97.9bp)	NFATC4/MA1525.1/Jaspar(0.627) More Information | Similar Motifs Found	motif file (matrix
11	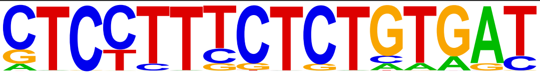	1e-49	-1.133e+02	1.43%	0.11%	53.2bp (52.8bp)	PRDM1/MA0508.3/Jaspar(0.665) More Information | Similar Motifs Found	motif file (matrix
12	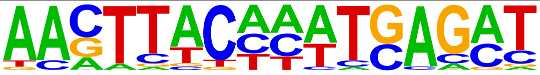	1e-46	-1.080e+02	0.51%	0.00%	47.2bp (11.3bp)	PB0058.1_Sfpi1_1/Jaspar(0.520) More Information | Similar Motifs Found	motif file (matrix
13	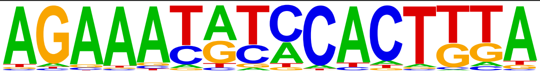	1e-45	-1.055e+02	0.70%	0.01%	51.7bp (82.0bp)	NKX2-8/MA0673.1/Jaspar(0.601) More Information | Similar Motifs Found	motif file (matrix
14	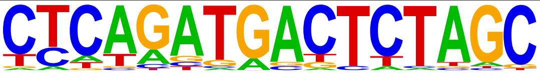	1e-43	-9.906e+01	1.12%	0.07%	47.9bp (49.7bp)	FOSL1/MA0477.2/Jaspar(0.536) More Information | Similar Motifs Found	motif file (matrix
15	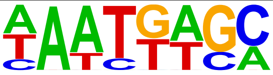	1e-42	-9.782e+01	29.11%	20.67%	56.6bp (64.7bp)	NOTO/MA0710.1/Jaspar(0.807) More Information | Similar Motifs Found	motif file (matrix
16	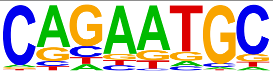	1e-40	-9.281e+01	28.63%	20.45%	55.8bp (63.6bp)	ZNF143/MA0088.2/Jaspar(0.691) More Information | Similar Motifs Found	motif file (matrix
17	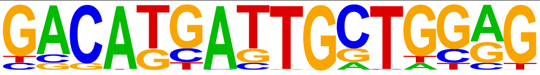	1e-39	-9.146e+01	0.51%	0.01%	52.1bp (34.9bp)	MEIS1(var.2)/MA1639.1/Jaspar(0.613) More Information | Similar Motifs Found	motif file (matrix
18	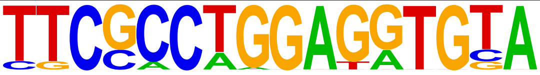	1e-33	-7.631e+01	0.38%	0.00%	36.3bp (0.0bp)	EBF1/MA0154.4/Jaspar(0.557) More Information | Similar Motifs Found	motif file (matrix
19	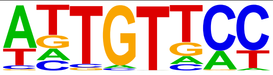	1e-32	-7.459e+01	18.48%	12.41%	55.6bp (62.1bp)	SOX8/MA0868.2/Jaspar(0.847) More Information | Similar Motifs Found	motif file (matrix
20	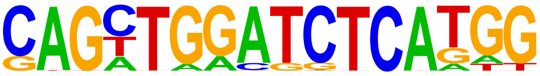	1e-29	-6.743e+01	0.80%	0.06%	63.4bp (47.0bp)	Ap4(bHLH)/AML-Tfap4-ChIP-Seq(GSE45738)/Homer(0.578) More Information | Similar Motifs Found	motif file (matrix
21	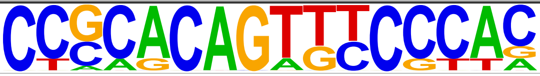	1e-28	-6.616e+01	0.34%	0.00%	58.3bp (14.3bp)	ZNF75D/MA1601.1/Jaspar(0.580) More Information | Similar Motifs	motif file

							Found	(matrix
22	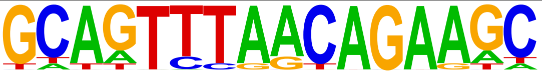	1e-28	-6.616e+01	0.34%	0.00%	41.9bp (0.0bp)	OSR2/MA1646.1/Jaspar(0.698) More Information | Similar Motifs Found	motif file (matrix
23	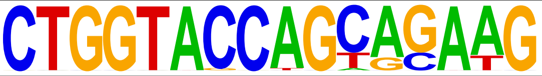	1e-28	-6.616e+01	0.34%	0.00%	37.8bp (2.4bp)	GCM1/MA0646.1/Jaspar(0.568) More Information | Similar Motifs Found	motif file (matrix
24	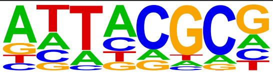	1e-28	-6.571e+01	52.62%	44.51%	56.2bp (68.7bp)	CEBPG/MA0838.1/Jaspar(0.702) More Information | Similar Motifs Found	motif file (matrix
25	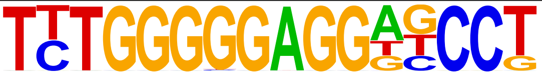	1e-26	-6.118e+01	0.32%	0.00%	42.9bp (32.3bp)	Znf281/MA1630.1/Jaspar(0.767) More Information | Similar Motifs Found	motif file (matrix
26	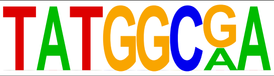	1e-23	-5.474e+01	0.80%	0.08%	56.0bp (68.1bp)	YY2/MA0748.2/Jaspar(0.711) More Information | Similar Motifs Found	motif file (matrix
27	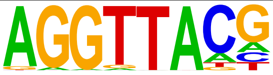	1e-17	-3.939e+01	5.49%	3.11%	53.3bp (60.8bp)	SIX1/MA1118.1/Jaspar(0.776) More Information | Similar Motifs Found	motif file (matrix
28	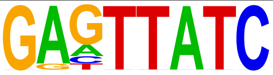	1e-16	-3.784e+01	2.55%	1.08%	54.3bp (64.0bp)	GATA5/MA0766.2/Jaspar(0.778) More Information | Similar Motifs Found	motif file (matrix
29	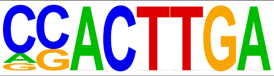	1e-14	-3.237e+01	3.57%	1.87%	52.5bp (66.1bp)	NKX2-8/MA0673.1/Jaspar(0.900) More Information | Similar Motifs Found	motif file (matrix
30 *	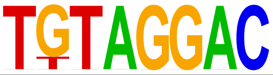	1e-11	-2.533e+01	1.12%	0.38%	55.7bp (55.7bp)	PH0065.1_Hoxc10/Jaspar(0.704) More Information | Similar Motifs Found	motif file (matrix
31 *	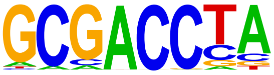	1e-10	-2.500e+01	4.98%	3.15%	55.2bp (69.6bp)	NR2C2(var.2)/MA1536.1/Jaspar(0.708) More Information | Similar Motifs Found	motif file (matrix
32 *	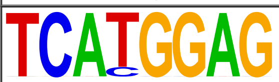	1e-8	-1.943e+01	1.58%	0.75%	61.8bp (58.9bp)	Pax2/MA0067.1/Jaspar(0.673) More Information | Similar Motifs Found	motif file (matrix
33 *	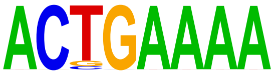	1e-8	-1.903e+01	2.11%	1.12%	57.2bp (64.2bp)	Hoxd12(Homeobox)/ChickenMSG-Hoxd12.Flag-ChIP-Seq(GSE86088)/Homer(0.711) More Information | Similar Motifs Found	motif file (matrix
34 *	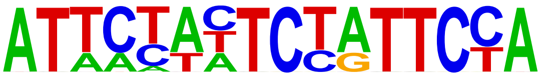	1e-6	-1.593e+01	0.11%	0.00%	39.4bp (0.0bp)	ZNF189(Zf)/HEK293-ZNF189.GFP-ChIP-Seq(GSE58341)/Homer(0.652) More Information | Similar Motifs Found	motif file (matrix

Homer *de novo* Motif Results (./MotifSearch/homer/Arcuate/)

Known Motif Enrichment Results

Gene Ontology Enrichment Results

If Homer is having trouble matching a motif to a known motif, try copy/pasting the matrix file into STAMP
More information on motif finding results: HOMER | Description of Results | Tips
Total target sequences = 3198

Total background sequences = 46280
* - possible false positive

Rank	Motif	P-value	log P-pvalue	% of Targets	% of Background	STD(Bg STD)	Best Match/Details	Motif File
1	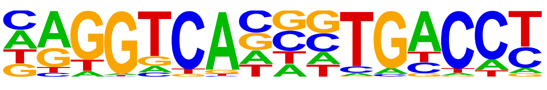	1e-1292	-2.977e+03	35.40%	1.18%	41.1bp (61.2bp)	ERE(NR),IR3/MCF7-ERa-ChIP-Seq(Unpublished)/Homer(0.977) More Information | Similar Motifs Found	motif file (matrix)
2	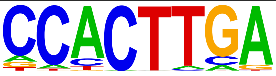	1e-84	-1.936e+02	18.07%	7.51%	57.2bp (61.7bp)	NKX2-3/MA0672.1/Jaspar(0.942) More Information | Similar Motifs Found	motif file (matrix)
3	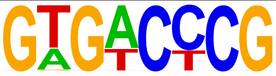	1e-74	-1.717e+02	29.89%	16.76%	54.3bp (65.2bp)	PB0153.1_Nr2f2_2/Jaspar(0.809) More Information | Similar Motifs Found	motif file (matrix)
4	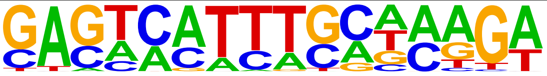	1e-68	-1.584e+02	3.94%	0.49%	55.0bp (64.2bp)	JDP2/MA0655.1/Jaspar(0.561) More Information | Similar Motifs Found	motif file (matrix)
5	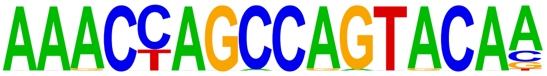	1e-66	-1.542e+02	2.28%	0.11%	40.1bp (59.2bp)	FOXA1:AR(Forkhead,NR)/LNCAP-AR-ChIP-Seq(GSE27824)/Homer(0.612) More Information | Similar Motifs Found	motif file (matrix)
6	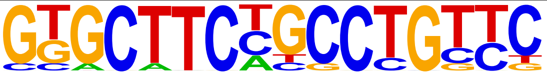	1e-64	-1.478e+02	3.03%	0.28%	57.0bp (63.5bp)	POL008.1_DCE_S_I/Jaspar(0.616) More Information | Similar Motifs Found	motif file (matrix)
7	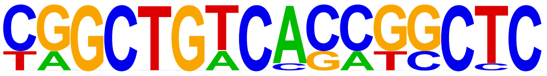	1e-59	-1.360e+02	2.25%	0.14%	54.4bp (54.5bp)	Meis1(Homeobox)/MastCells-Meis1-ChIP-Seq(GSE48085)/Homer(0.694) More Information | Similar Motifs Found	motif file (matrix)
8	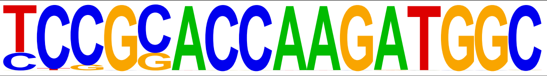	1e-58	-1.337e+02	2.50%	0.20%	59.0bp (70.9bp)	YY1/MA0095.2/Jaspar(0.713) More Information | Similar Motifs Found	motif file (matrix)
9	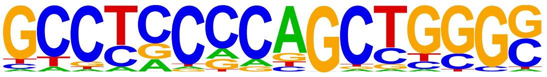	1e-55	-1.267e+02	2.50%	0.22%	57.3bp (70.0bp)	Zic2(Zf)/ESC-Zic2-ChIP-Seq(SRP197560)/Homer(0.691) More Information | Similar Motifs Found	motif file (matrix)
10	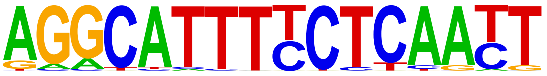	1e-53	-1.234e+02	2.63%	0.26%	59.5bp (55.0bp)	PU.1(ETS)/ThioMac-PU.1-ChIP-Seq(GSE21512)/Homer(0.596) More Information | Similar Motifs Found	motif file (matrix)
11	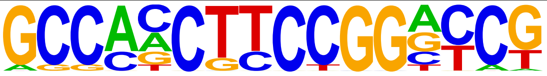	1e-52	-1.208e+02	2.53%	0.24%	54.1bp (68.2bp)	ZBTB7A/MA0750.2/Jaspar(0.729) More Information | Similar Motifs Found	motif file (matrix)
12	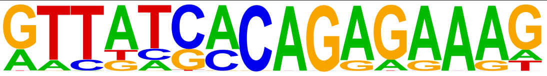	1e-52	-1.205e+02	1.88%	0.11%	47.5bp (59.8bp)	PRDM1/MA0508.3/Jaspar(0.687) More Information | Similar Motifs Found	motif file (matrix)
13	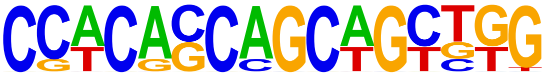	1e-50	-1.152e+02	2.35%	0.21%	50.5bp (73.3bp)	MyoD(bHLH)/Myotube-MyoD-ChIP-Seq(GSE21614)/Homer(0.682) More Information | Similar Motifs Found	motif file (matrix)
14	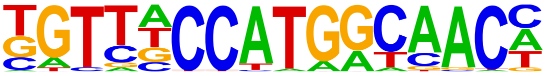	1e-48	-1.116e+02	2.10%	0.17%	52.1bp (53.8bp)	Rfx2(HTH)/LoVo-RFX2-ChIP-Seq(GSE49402)/Homer(0.948) More Information | Similar Motifs Found	motif file (matrix)
15	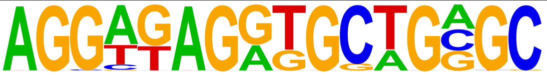	1e-45	-1.038e+02	2.41%	0.27%	50.4bp (73.7bp)	MYOD1/MA0499.2/Jaspar(0.570) More Information | Similar Motifs Found	motif file (matrix)
16	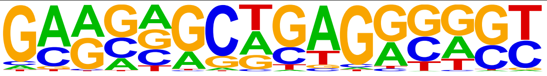	1e-44	-1.028e+02	2.28%	0.24%	50.6bp (66.6bp)	ZNF460/MA1596.1/Jaspar(0.550) More Information | Similar Motifs Found	motif file (matrix)
17	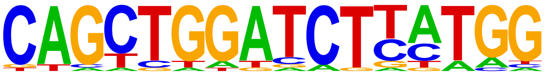	1e-33	-7.600e+01	1.69%	0.18%	54.9bp (57.6bp)	Tcf21(bHLH)/ArterySmoothMuscle-Tcf21-ChIP-Seq(GSE61369)/Homer(0.638) More Information | Similar Motifs Found	motif file (matrix)
18	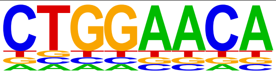	1e-31	-7.335e+01	4.16%	1.22%	54.7bp (64.4bp)	ZBTB12/MA1649.1/Jaspar(0.903) More Information | Similar Motifs Found	motif file (matrix)
19	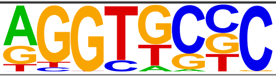	1e-28	-6.566e+01	3.72%	1.10%	57.8bp (60.2bp)	Slug(Zf)/Mesoderm-Snai2-ChIP-Seq(GSE61475)/Homer(0.714)	motif file (matrix)

							More Information | Similar Motifs Found	
20	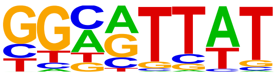	1e-26	-5.995e+01	4.75%	1.76%	56.7bp (60.4bp)	Pitx1(Homeobox)/Chicken-Pitx1-ChIP-Seq(GSE38910)/Homer(0.736) More Information | Similar Motifs Found	motif file (matrix)
21	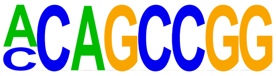	1e-25	-5.913e+01	3.00%	0.81%	56.6bp (63.4bp)	Zic1::Zic2/MA1628.1/Jaspar(0.797) More Information | Similar Motifs Found	motif file (matrix)
22	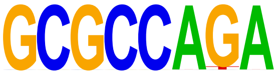	1e-25	-5.797e+01	2.94%	0.80%	52.3bp (69.2bp)	E2F4/MA0470.2/Jaspar(0.770) More Information | Similar Motifs Found	motif file (matrix)
23	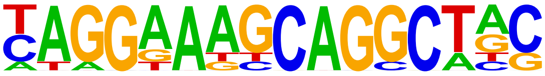	1e-20	-4.779e+01	1.16%	0.14%	56.6bp (45.7bp)	TEAD1(TEAD)/HepG2-TEAD1-ChIP-Seq(Encode)/Homer(0.574) More Information | Similar Motifs Found	motif file (matrix)
24	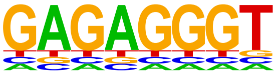	1e-20	-4.732e+01	3.35%	1.16%	50.6bp (61.6bp)	PB0114.1_Egr1_2/Jaspar(0.671) More Information | Similar Motifs Found	motif file (matrix)
25	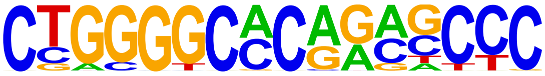	1e-18	-4.282e+01	1.16%	0.17%	46.7bp (52.2bp)	ZNF692(Zf)/HEK293-ZNF692.GFP-ChIP-Seq(GSE58341)/Homer(0.642) More Information | Similar Motifs Found	motif file (matrix)
26	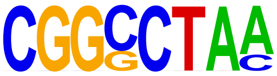	1e-18	-4.229e+01	2.91%	0.99%	55.4bp (61.5bp)	ZNF711(Zf)/SHSY5Y-ZNF711-ChIP-Seq(GSE20673)/Homer(0.733) More Information | Similar Motifs Found	motif file (matrix)
27	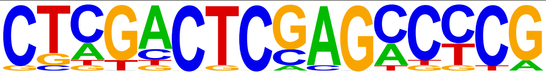	1e-14	-3.402e+01	1.09%	0.20%	49.9bp (47.9bp)	ZBTB6/MA1581.1/Jaspar(0.581) More Information | Similar Motifs Found	motif file (matrix)
28	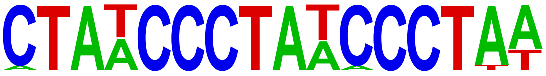	1e-13	-3.205e+01	0.25%	0.00%	51.5bp (0.0bp)	PSE(SNAPc)/K562-mStart-Seq/Homer(0.553) More Information | Similar Motifs Found	motif file (matrix)
29 *	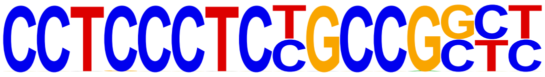	1e-4	-1.121e+01	0.13%	0.01%	56.5bp (38.8bp)	ZSCAN22(Zf)/HEK293-ZSCAN22.GFP-ChIP-Seq(GSE58341)/Homer(0.624) More Information | Similar Motifs Found	motif file (matrix)
30 *	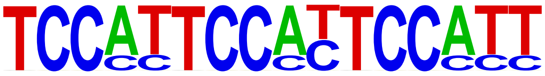	1e-4	-9.862e+00	0.09%	0.00%	49.8bp (0.0bp)	TEAD1(TEAD)/HepG2-TEAD1-ChIP-Seq(Encode)/Homer(0.596) More Information | Similar Motifs Found	motif file (matrix)

Homer *de novo* Motif Results (./MotifSearch/homer/Common/)

Known Motif Enrichment Results
Gene Ontology Enrichment Results
If Homer is having trouble matching a motif to a known motif, try copy/pasting the matrix file into STAMP
More information on motif finding results: HOMER | Description of Results | Tips
Total target sequences = 3413
Total background sequences = 45087
* - possible false positive

Rank	Motif	P-value	log P-pvalue	% of Targets	% of Background	STD(Bg STD)	Best Match/Details
1	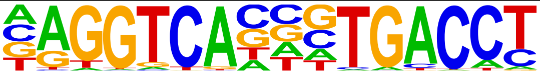	1e-1478	-3.406e+03	25.81%	0.23%	40.2bp (60.4bp)	ESR1/MA0112.3/Jaspar(0.974) More Information | Similar Motifs Found
2	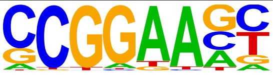	1e-145	-3.348e+02	16.61%	4.75%	54.4bp (65.6bp)	Elk4(ETS)/Hela-Elk4-ChIP-Seq(GSE31477)/Homer(0.973) More Information | Similar Motifs Found
3	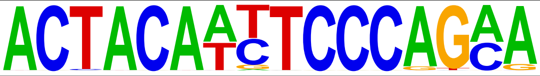	1e-125	-2.887e+02	5.07%	0.40%	50.8bp (65.7bp)	THAP11/MA1573.1/Jaspar(0.955) More Information | Similar Motifs Found
4	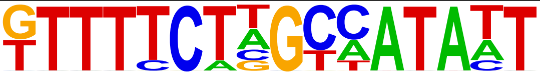	1e-73	-1.700e+02	0.97%	0.00%	58.0bp (0.0bp)	E2F7/MA0758.1/Jaspar(0.622) More Information | Similar Motifs Found
5	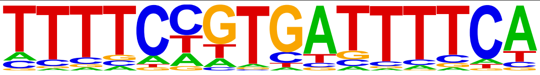	1e-65	-1.518e+02	0.88%	0.00%	43.8bp (0.0bp)	GFI1/MA0038.2/Jaspar(0.640) More Information | Similar Motifs Found
6	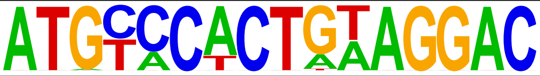	1e-60	-1.399e+02	0.82%	0.00%	52.9bp (0.0bp)	ZKSCAN1(Zf)/HepG2-ZKSCAN1-ChIP-Seq(Encode)/Homer(0.559) More Information | Similar Motifs Found
7	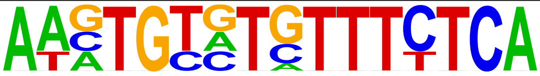	1e-55	-1.281e+02	0.76%	0.00%	49.8bp (0.0bp)	Stat2/MA1623.1/Jaspar(0.609) More Information | Similar Motifs Found
8	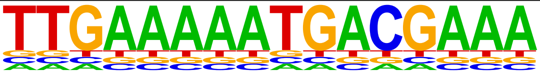	1e-45	-1.051e+02	0.64%	0.00%	54.1bp (0.0bp)	FOSL1::JUND(var.2)/MA1143.1/Jaspar(0.614) More Information | Similar Motifs Found
9	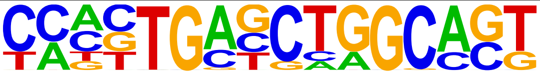	1e-45	-1.051e+02	0.64%	0.00%	46.6bp (12.7bp)	HIC1(Zf)/Treg-ZBTB29-ChIP-Seq(GSE99889)/Homer(0.623) More Information | Similar Motifs Found
10	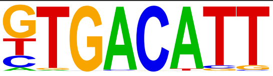	1e-39	-9.000e+01	17.81%	10.33%	53.8bp (62.8bp)	MEIS1/MA0498.2/Jaspar(0.821) More Information | Similar Motifs Found
11	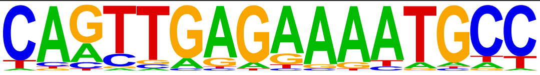	1e-38	-8.937e+01	2.26%	0.31%	56.4bp (52.7bp)	MYB/MA0100.3/Jaspar(0.566) More Information | Similar Motifs Found
12	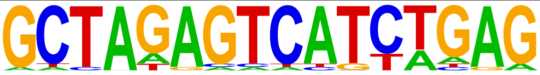	1e-36	-8.368e+01	1.05%	0.04%	46.7bp (45.8bp)	USF1/MA0093.3/Jaspar(0.535) More Information | Similar Motifs Found
13	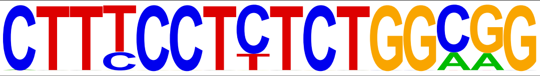	1e-35	-8.270e+01	0.53%	0.00%	49.3bp (7.9bp)	T1ISRE(IRF)/ThioMac-Ifnb-Expression/Homer(0.635) More Information | Similar Motifs Found
14	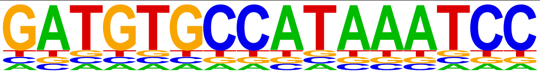	1e-33	-7.724e+01	0.50%	0.00%	35.8bp (0.0bp)	Hoxc9(Homeobox)/Ainv15-Hoxc9-ChIP-Seq(GSE21812)/Homer(0.703) More Information | Similar Motifs Found
15	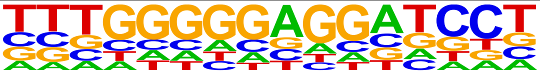	1e-33	-7.724e+01	0.50%	0.00%	43.4bp (0.0bp)	Znf281/MA1630.1/Jaspar(0.780) More Information | Similar Motifs Found
16	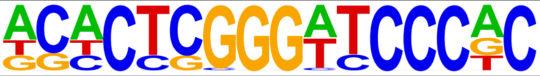	1e-33	-7.724e+01	0.50%	0.00%	40.5bp (40.9bp)	TFDP1/MA1122.1/Jaspar(0.588) More Information | Similar Motifs Found
17	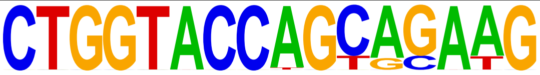	1e-33	-7.724e+01	0.50%	0.00%	40.0bp (14.7bp)	GCM1/MA0646.1/Jaspar(0.574) More Information | Similar Motifs Found
18	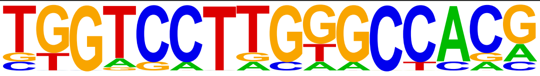	1e-32	-7.512e+01	0.56%	0.01%	33.7bp (39.4bp)	ZBTB6/MA1581.1/Jaspar(0.693) More Information | Similar Motifs Found
19	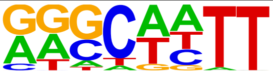	1e-31	-7.212e+01	38.34%	28.98%	57.2bp (62.9bp)	ISL2/MA0914.1/Jaspar(0.756) More Information | Similar Motifs Found
20	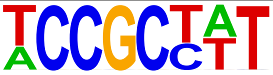	1e-30	-7.099e+01	19.68%	12.61%	54.0bp (70.3bp)	YY2/MA0748.2/Jaspar(0.798) More Information | Similar Motifs Found
21	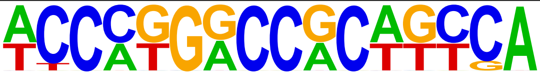	1e-30	-7.030e+01	0.53%	0.00%	44.4bp (48.9bp)	Klf4(Zf)/mES-Klf4-ChIP-Seq(GSE11431)/Homer(0.583) More Information | Similar Motifs Found
22	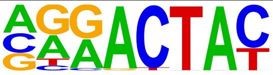	1e-29	-6.741e+01	6.50%	2.77%	52.2bp (64.2bp)	THAP11/MA1573.1/Jaspar(0.642) More Information | Similar Motifs Found
23	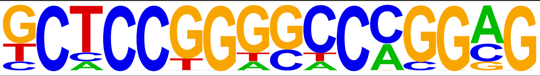	1e-28	-6.649e+01	0.44%	0.00%	34.0bp (0.0bp)	POL013.1_MED-1/Jaspar(0.583) More Information | Similar Motifs Found

24	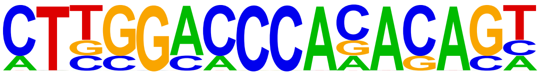	1e-28	-6.553e+01	0.50%	0.00%	31.6bp (30.6bp)	ZNF692(Zf)/HEK293-ZNF692.GFP-ChIP-Seq(GSE58341)/Homer(0.648) More Information | Similar Motifs Found
25	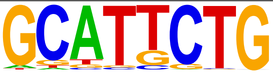	1e-25	-5.975e+01	11.51%	6.56%	55.3bp (63.3bp)	ZNF143|STAF(Zf)/CUTLL-ZNF143-ChIP-Seq(GSE29600)/Homer(0.676) More Information | Similar Motifs Found
26	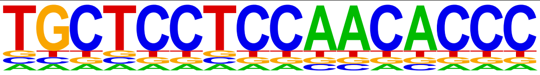	1e-24	-5.600e+01	0.38%	0.00%	38.8bp (7.9bp)	Wt1/MA1627.1/Jaspar(0.623) More Information | Similar Motifs Found
27	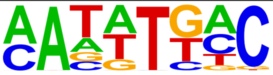	1e-22	-5.291e+01	21.29%	14.89%	56.1bp (63.8bp)	NEUROG2/MA0669.1/Jaspar(0.685) More Information | Similar Motifs Found
28	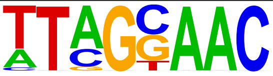	1e-17	-4.047e+01	11.57%	7.39%	55.6bp (62.9bp)	RFX7/MA1554.1/Jaspar(0.845) More Information | Similar Motifs Found
29	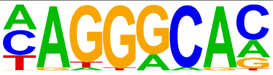	1e-15	-3.604e+01	14.62%	10.16%	54.3bp (58.9bp)	ERRg(NR)/Kidney-ESRRG-ChIP-Seq(GSE104905)/Homer(0.810) More Information | Similar Motifs Found
30	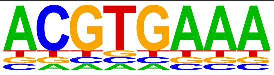	1e-12	-2.832e+01	0.97%	0.20%	61.7bp (62.6bp)	HIF2a(bHLH)/785_O-HIF2a-ChIP-Seq(GSE34871)/Homer(0.759) More Information | Similar Motifs Found
31 *	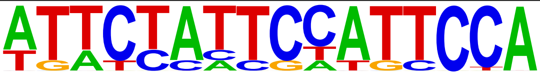	1e-8	-1.900e+01	0.21%	0.01%	46.5bp (13.4bp)	TEAD3(TEA)/HepG2-TEAD3-ChIP-Seq(Encode)/Homer(0.666) More Information | Similar Motifs Found
32 *	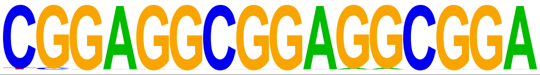	1e-6	-1.428e+01	0.15%	0.01%	33.2bp (10.6bp)	ZNF460/MA1596.1/Jaspar(0.716) More Information | Similar Motifs Found

Homer *de novo* Motif Results (./MotifSearch/homer/AVPV/)

[Known Motif Enrichment Results](#)
[Gene Ontology Enrichment Results](#)
If Homer is having trouble matching a motif to a known motif, try copy/pasting the matrix file into [STAMP](#)
More information on motif finding results: [HOMER](#) | [Description of Results](#) | [Tips](#)
Total target sequences = 1385

Total background sequences = 46326
* - possible false positive

Rank	Motif	P-value	log P-pvalue	% of Targets	% of Background	STD(Bg STD)	Best Match/Details
1	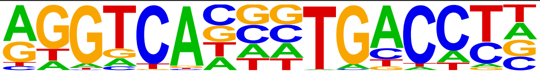	1e-569	-1.310e+03	33.29%	0.89%	39.8bp (56.8bp)	ERE(NR),IR3/MCF7-ERa-ChIP-Seq(Unpublished)/Homer(0.974) More Information | Similar Motifs Found
2	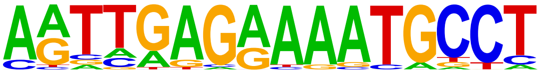	1e-50	-1.165e+02	4.19%	0.23%	63.9bp (52.6bp)	PU.1(ETS)/ThioMac-PU.1-ChIP-Seq(GSE21512)/Homer(0.592) More Information | Similar Motifs Found
3	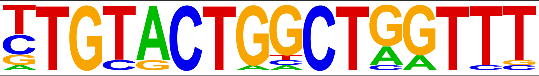	1e-48	-1.126e+02	2.74%	0.06%	42.7bp (45.6bp)	FOXA1:AR(Forkhead,NR)/LNCAP-AR-ChIP-Seq(GSE27824)/Homer(0.625) More Information | Similar Motifs Found
4	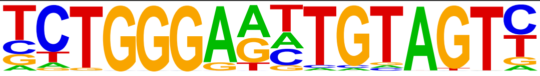	1e-43	-9.988e+01	3.97%	0.27%	49.7bp (62.0bp)	THAP11/MA1573.1/Jaspar(0.960) More Information | Similar Motifs Found
5	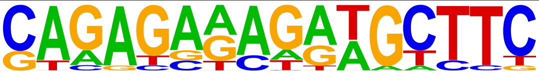	1e-41	-9.460e+01	2.96%	0.12%	47.5bp (45.3bp)	PRDM1/MA0508.3/Jaspar(0.600) More Information | Similar Motifs Found
6	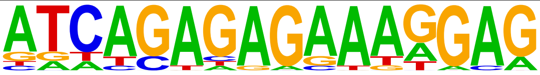	1e-34	-7.871e+01	3.03%	0.20%	54.5bp (53.4bp)	PRDM1/MA0508.3/Jaspar(0.692) More Information | Similar Motifs Found
7	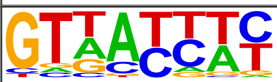	1e-33	-7.702e+01	36.25%	21.89%	55.6bp (62.4bp)	GATA3(Zf)/iTreg-Gata3-ChIP-Seq(GSE20898)/Homer(0.679) More Information | Similar Motifs Found
8	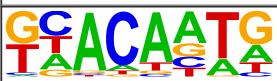	1e-28	-6.660e+01	37.98%	24.32%	52.9bp (63.2bp)	SOX13/MA1120.1/Jaspar(0.871) More Information | Similar Motifs Found
9	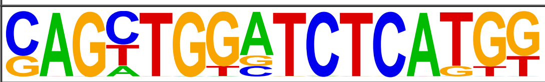	1e-27	-6.412e+01	1.59%	0.04%	66.9bp (35.7bp)	Tcf21(bHLH)/ArterySmoothMuscle-Tcf21-ChIP-Seq(GSE61369)/Homer(0.579) More Information | Similar Motifs Found
10	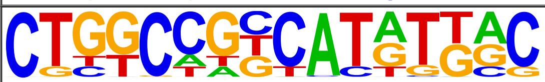	1e-24	-5.624e+01	0.79%	0.00%	66.8bp (0.0bp)	YY1(Zf)/Promoter/Homer(0.663) More Information | Similar Motifs Found
11	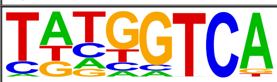	1e-22	-5.251e+01	20.36%	11.12%	52.1bp (63.0bp)	NR2C1/MA1535.1/Jaspar(0.815) More Information | Similar Motifs Found
12	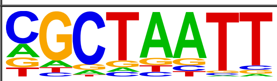	1e-22	-5.081e+01	44.62%	32.02%	56.7bp (64.3bp)	GBX1/MA0889.1/Jaspar(0.852) More Information | Similar Motifs Found
13	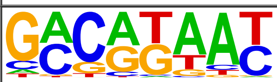	1e-20	-4.744e+01	23.18%	13.74%	55.9bp (63.7bp)	FOSL1::JUND(var.2)/MA1143.1/Jaspar(0.658) More Information | Similar Motifs Found
14	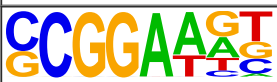	1e-17	-4.132e+01	15.45%	8.26%	55.0bp (66.6bp)	Elk4(ETS)/Hela-Elk4-ChIP-Seq(GSE31477)/Homer(0.961) More Information | Similar Motifs Found
15	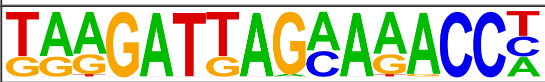	1e-16	-3.877e+01	0.58%	0.00%	39.9bp (5.2bp)	GATA1/MA0035.4/Jaspar(0.701) More Information | Similar Motifs Found
16	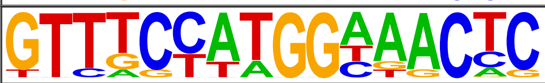	1e-16	-3.815e+01	1.16%	0.05%	52.9bp (55.8bp)	Rfx2(HTH)/LoVo-RFX2-ChIP-Seq(GSE49402)/Homer(0.874) More Information | Similar Motifs Found
17	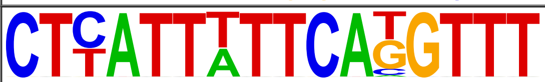	1e-14	-3.325e+01	0.58%	0.01%	49.2bp (0.0bp)	SIX1/MA1118.1/Jaspar(0.608) More Information | Similar Motifs Found
18 *	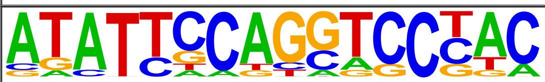	1e-11	-2.724e+01	1.01%	0.07%	56.8bp (55.6bp)	TEAD1/MA0090.3/Jaspar(0.645) More Information | Similar Motifs Found
19 *	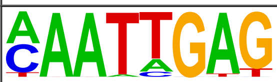	1e-10	-2.470e+01	11.91%	6.95%	54.2bp (66.1bp)	Hnf6b(Homeobox)/LNCaP-Hnf6b-ChIP-Seq(GSE106305)/Homer(0.732) More Information | Similar Motifs Found
20 *	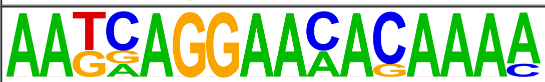	1e-9	-2.240e+01	0.36%	0.00%	55.2bp (0.0bp)	IKZF1/MA1508.1/Jaspar(0.610) More Information | Similar Motifs Found
21 *	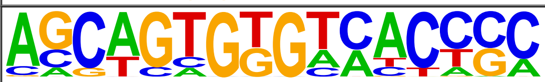	1e-9	-2.240e+01	0.36%	0.00%	46.0bp (0.0bp)	ZNF75D/MA1601.1/Jaspar(0.563) More Information | Similar Motifs Found
22 *	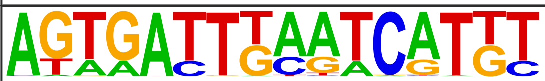	1e-9	-2.232e+01	0.58%	0.02%	54.9bp (22.9bp)	PH0014.1_Cphx/Jaspar(0.713) More Information | Similar Motifs Found
23 *	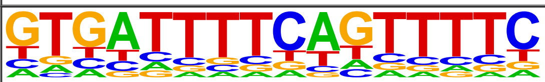	1e-9	-2.173e+01	0.65%	0.03%	61.4bp (82.1bp)	STAT1::STAT2/MA0517.1/Jaspar(0.727) More Information | Similar Motifs Found

24 *	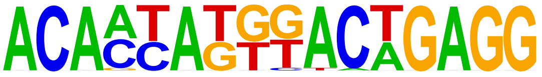	1e-9	-2.117e+01	0.43%	0.01%	48.1bp (3.5bp)	TFAP2A/MA0003.4/Jaspar(0.546) More Information | Similar Motifs Found
25 *	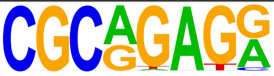	1e-8	-1.880e+01	6.86%	3.64%	56.3bp (71.7bp)	HINFP/MA0131.2/Jaspar(0.629) More Information | Similar Motifs Found
26 *	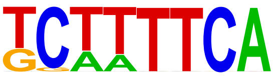	1e-7	-1.755e+01	11.19%	7.11%	57.0bp (66.7bp)	Hoxd10(Homeobox)/ChickenMSG-Hoxd10.Flag-ChIP-Seq(GSE86088)/Homer(0.682) More Information | Similar Motifs Found
27 *	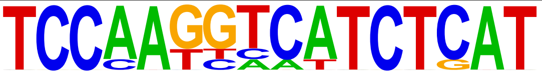	1e-7	-1.727e+01	0.29%	0.00%	57.1bp (0.0bp)	PB0014.1_Esrra_1/Jaspar(0.631) More Information | Similar Motifs Found
28 *	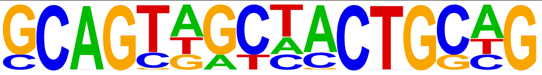	1e-7	-1.695e+01	0.36%	0.01%	55.1bp (0.0bp)	PRDM9(Zf)/Testis-DMC1-ChIP-Seq(GSE35498)/Homer(0.614) More Information | Similar Motifs Found
29 *	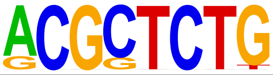	1e-6	-1.485e+01	3.68%	1.68%	51.0bp (69.3bp)	ZNF768(Zf)/Rajj-ZNF768-ChIP-Seq(GSE111879)/Homer(0.686) More Information | Similar Motifs Found
30 *	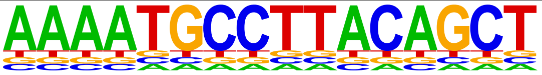	1e-4	-9.264e+00	91.70%	88.60%	54.3bp (60.4bp)	PH0158.1_Rhox11_2/Jaspar(0.582) More Information | Similar Motifs Found
31 *	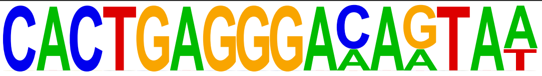	1e-3	-7.744e+00	0.14%	0.00%	67.2bp (0.0bp)	PKNOX1/MA0782.2/Jaspar(0.609) More Information | Similar Motifs Found

Homer *de novo* Motif Results (./MotifSearch/homer/Mammary/)

Known Motif Enrichment Results

Gene Ontology Enrichment Results

If Homer is having trouble matching a motif to a known motif, try copy/pasting the matrix file into STAMP
More information on motif finding results: HOMER | Description of Results | Tips
Total target sequences = 12505

Total background sequences = 37090
* - possible false positive

Rank	Motif	P-value	log P-pvalue	% of Targets	% of Background	STD(Bg STD)	Best Match/Details	Motif File
1	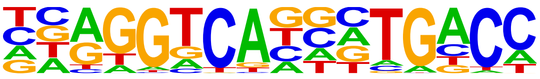	1e-3103	-7.146e+03	42.65%	5.73%	37.5bp (63.8bp)	ERE(NR),IR3/MCF7-ERa-ChIP-Seq(Unpublished)/Homer(0.979) More Information | Similar Motifs Found	motif file (matri
2	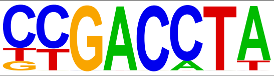	1e-732	-1.686e+03	28.38%	9.90%	49.7bp (66.4bp)	RORA(var.2)/MA0072.1/Jaspar(0.769) More Information | Similar Motifs Found	motif file (matri
3	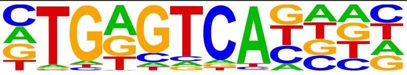	1e-621	-1.430e+03	29.19%	11.50%	51.8bp (63.7bp)	Smad2::Smad3/MA1622.1/Jaspar(0.938) More Information | Similar Motifs Found	motif file (matri
4	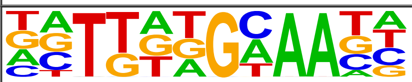	1e-509	-1.174e+03	21.17%	7.51%	51.1bp (61.6bp)	CEBPD/MA0836.2/Jaspar(0.876) More Information | Similar Motifs Found	motif file (matri
5	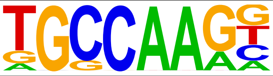	1e-260	-6.003e+02	41.90%	27.51%	55.8bp (63.4bp)	NFIC/MA0161.2/Jaspar(0.966) More Information | Similar Motifs Found	motif file (matri
6	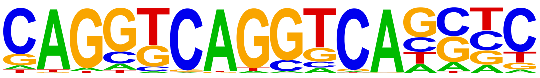	1e-182	-4.210e+02	15.34%	7.63%	49.7bp (62.7bp)	THRb(NR)/Liver-NR1A2-ChIP-Seq(GSE52613)/Homer(0.681) More Information | Similar Motifs Found	motif file (matri
7	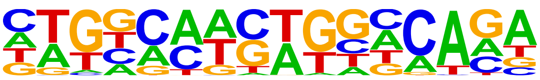	1e-161	-3.728e+02	26.67%	16.97%	53.1bp (61.8bp)	Bcl11a(Zf)/HSPC-BCL11A-ChIP-Seq(GSE104676)/Homer(0.726) More Information | Similar Motifs Found	motif file (matri
8	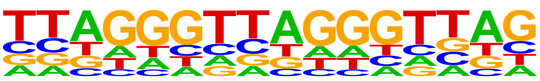	1e-153	-3.546e+02	0.72%	0.01%	39.6bp (42.2bp)	PSE(SNAPc)/K562-mStart-Seq/Homer(0.676) More Information | Similar Motifs Found	motif file (matri
9	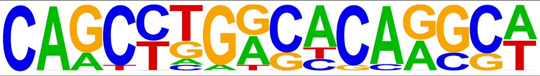	1e-151	-3.494e+02	1.68%	0.13%	55.1bp (14.0bp)	PB0206.1_Zic2_2/Jaspar(0.554) More Information | Similar Motifs Found	motif file (matri
10	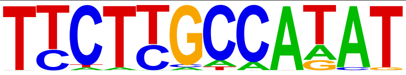	1e-149	-3.448e+02	0.70%	0.01%	54.1bp (42.8bp)	E2F8/MA0865.1/Jaspar(0.657) More Information | Similar Motifs Found	motif file (matri
11	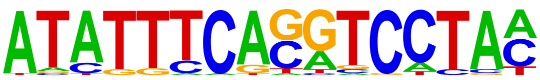	1e-139	-3.205e+02	0.66%	0.01%	51.2bp (69.7bp)	PU.1:IRF8(ETS:IRF)/pDC-Irf8-ChIP-Seq(GSE66899)/Homer(0.616) More Information | Similar Motifs Found	motif file (matri
12	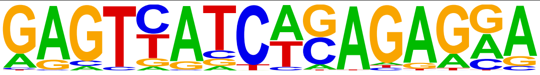	1e-133	-3.085e+02	2.45%	0.39%	41.8bp (58.5bp)	FOSL1::JUN/MA1128.1/Jaspar(0.565) More Information | Similar Motifs Found	motif file (matri
13	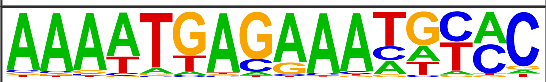	1e-122	-2.828e+02	0.66%	0.01%	51.7bp (37.0bp)	PB0148.1_Mtf1_2/Jaspar(0.633) More Information | Similar Motifs Found	motif file (matri
14	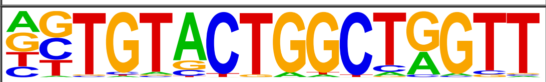	1e-120	-2.782e+02	1.93%	0.26%	35.6bp (63.1bp)	PB0060.1_Smad3_1/Jaspar(0.584) More Information | Similar Motifs Found	motif file (matri
15	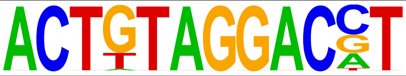	1e-118	-2.729e+02	0.58%	0.01%	49.8bp (13.2bp)	PH0065.1_Hoxc10/Jaspar(0.622) More Information | Similar Motifs Found	motif file (matri
16	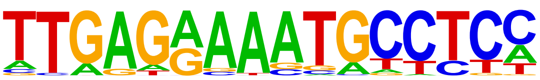	1e-118	-2.723e+02	2.30%	0.40%	58.3bp (54.7bp)	PU.1(ETS)/ThioMac-PU.1-ChIP-Seq(GSE21512)/Homer(0.598) More Information | Similar Motifs Found	motif file (matri
17	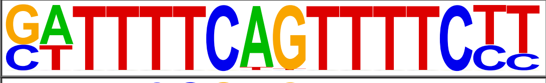	1e-117	-2.696e+02	0.63%	0.01%	54.5bp (6.1bp)	Stat2/MA1623.1/Jaspar(0.712) More Information | Similar Motifs Found	motif file (matri
18	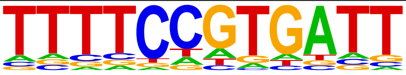	1e-107	-2.472e+02	0.63%	0.01%	54.3bp (50.3bp)	GFI1/MA0038.2/Jaspar(0.710) More Information | Similar Motifs Found	motif file (matri
19	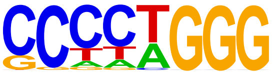	1e-98	-2.266e+02	15.21%	9.28%	55.7bp (61.1bp)	EBF1(EBF)/Near-E2A-ChIP-Seq(GSE21512)/Homer(0.920) More Information | Similar Motifs Found	motif file (matri
20	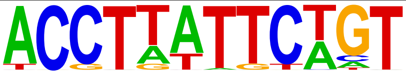	1e-92	-2.131e+02	0.48%	0.01%	62.5bp (45.1bp)	PH0152.1_Pou6f1_2/Jaspar(0.630) More Information | Similar Motifs Found	motif file (matri
21	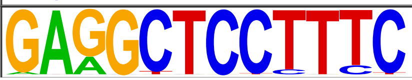	1e-92	-2.123e+02	1.52%	0.22%	48.9bp (57.8bp)	PB0137.1_Irf3_2/Jaspar(0.673) More Information | Similar Motifs	motif file

							Found	(matri
22	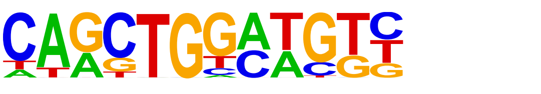	1e-82	-1.904e+02	2.35%	0.59%	59.3bp (62.7bp)	HEB(bHLH)/mES-Heb-ChIP-Seq(GSE53233)/Homer(0.762) More Information | Similar Motifs Found	motif file (matri
23	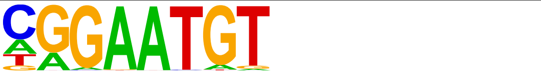	1e-74	-1.725e+02	9.11%	5.11%	54.1bp (62.9bp)	TEAD2/MA1121.1/Jaspar(0.937) More Information | Similar Motifs Found	motif file (matri
24	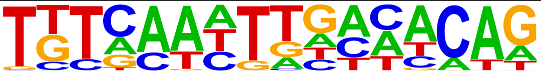	1e-63	-1.462e+02	12.23%	7.86%	55.0bp (61.2bp)	LIN54/MA0619.1/Jaspar(0.577) More Information | Similar Motifs Found	motif file (matri
25	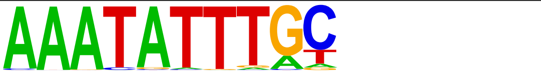	1e-56	-1.308e+02	3.26%	1.33%	54.4bp (57.3bp)	Arid5a/MA0602.1/Jaspar(0.770) More Information | Similar Motifs Found	motif file (matri
26	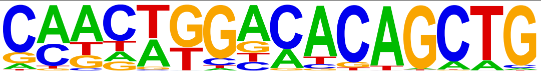	1e-56	-1.307e+02	1.68%	0.44%	56.1bp (60.1bp)	Ptf1a(var.2)/MA1619.1/Jaspar(0.779) More Information | Similar Motifs Found	motif file (matri
27	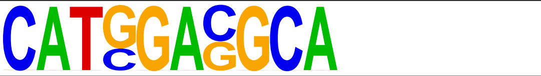	1e-41	-9.458e+01	1.14%	0.29%	62.7bp (62.4bp)	YY2/MA0748.2/Jaspar(0.587) More Information | Similar Motifs Found	motif file (matri
28	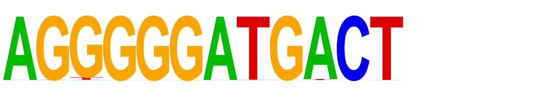	1e-28	-6.491e+01	0.19%	0.01%	56.4bp (5.8bp)	ZNF669(Zf)/HEK293-ZNF669.GFP-ChIP-Seq(GSE58341)/Homer(0.679) More Information | Similar Motifs Found	motif file (matri
29	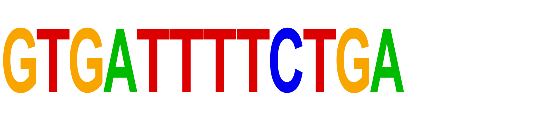	1e-21	-4.920e+01	0.18%	0.01%	54.8bp (95.8bp)	EWS:ERG-fusion(ETS)/CADO_ES1-EWS:ERG-ChIP-Seq(SRA014231)/Homer(0.647) More Information | Similar Motifs Found	motif file (matri
30	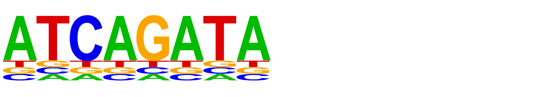	1e-19	-4.579e+01	1.09%	0.44%	56.7bp (54.7bp)	GATA3(Zf)/iTreg-Gata3-ChIP-Seq(GSE20898)/Homer(0.705) More Information | Similar Motifs Found	motif file (matri
31	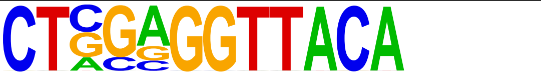	1e-17	-4.085e+01	0.14%	0.01%	53.1bp (3.9bp)	RUNX3/MA0684.2/Jaspar(0.619) More Information | Similar Motifs Found	motif file (matri
32	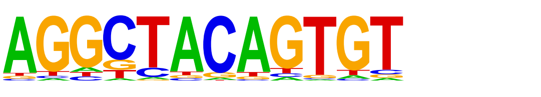	1e-13	-3.134e+01	0.11%	0.01%	57.7bp (49.7bp)	DMRT6(DM)/Testis-DMRT6-ChIP-Seq(GSE60440)/Homer(0.694) More Information | Similar Motifs Found	motif file (matri
33 *	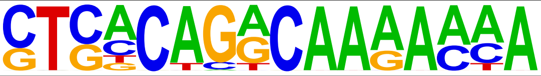	1e-7	-1.830e+01	0.09%	0.01%	57.8bp (0.0bp)	ZNF384/MA1125.1/Jaspar(0.601) More Information | Similar Motifs Found	motif file (matri
34 *	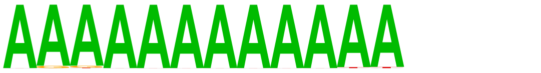	1e0	-2.232e+00	2.41%	2.24%	54.8bp (61.7bp)	ZNF384/MA1125.1/Jaspar(0.842) More Information | Similar Motifs Found	motif file (matri
